# D-Ring-Modified Analogues of Luotonin A with Reduced Planarity: Design, Synthesis, and Evaluation of Their Topoisomerase Inhibition-Associated Cytotoxicity

**DOI:** 10.1155/2019/2514524

**Published:** 2019-11-13

**Authors:** Abdulrahman I. Almansour, Raju Suresh Kumar, Natarajan Arumugam, Giulia Bianchini, J. Carlos Menéndez, Faruq Mohammad, Kotresha Dupadahalli, Mohammad Altaf

**Affiliations:** ^1^Department of Chemistry, College of Science, King Saud University, P. O. Box 2455, Riyadh 11451, Saudi Arabia; ^2^Unidad de Química Orgánica y Farmacéutica, Departamento de Química en Ciencias Farmacéuticas, Facultad de Farmacia, Universidad Complutense, 28040 Madrid, Spain; ^3^Surfactants Research Chair, Department of Chemistry, College of Science, King Saud University, P. O. Box 2455, Riyadh 11451, Saudi Arabia; ^4^Department of Botany, Davangere University, Shivagangothri, Davangere 577007, Karnataka, India; ^5^Central Laboratory, College of Science, King Saud University, P. O. Box 2455, Riyadh 11451, Saudi Arabia

## Abstract

A- and D-ring-modified luotonin-inspired heterocycles have been synthesized and were evaluated for their activity against the viability of four cancer cell lines in vitro, namely, MCF7, HCT116, JURKAT, and NCI-H460. The analysis of results indicated that two of the synthesized derivatives displayed good inhibition against the growth of the human colon cancer HCT116 cell line, with potencies lower than but in the same order of magnitude as camptothecin (CPT). These two luotonin analogues also showed an activity similar to that of the highly potent alkaloid CPT as inhibitors of topoisomerase I and also inhibited topoisomerase II. These results show that complete planarity is not a strict requirement for topoisomerase inhibition by luotonin-related compounds, paving the way to the design of analogues with improved solubility.

## 1. Introduction

Cancer and related diseases are mainly caused by a number of genetic, environmental, and lifestyle-associated factors and the disease is characterized by a shift in the controlled mechanisms that govern cell proliferation and differentiation, and normal physiological activity [1]. Cancerous diseases are life-threatening and continue to be the leading causes of death, surpassing cardiovascular disease. In 2012, there were 14.1 million new cases of cancer worldwide and 8.2 million cancer-related deaths, and it is estimated that about 32.6 million patients have survived after 5 years of a cancer diagnosis [2]. Therefore, the development of novel, more effective anticancer agents with good pharmacokinetic profiles remains an important and challenging goal in medicinal chemistry [3]. The NIH (National Institutes of Health) database describes a large number of anticancer compounds, organized according to their targets and mechanisms of action [4]. Polycyclic nitrogen heterocycles are interesting pharmacophores in this regard as they can interfere with the functioning of several DNA-associated enzymes, including the topoisomerases, in a process that is normally initiated by compound intercalation between the base pairs of double-stranded DNA [5].

**Figure 1:**
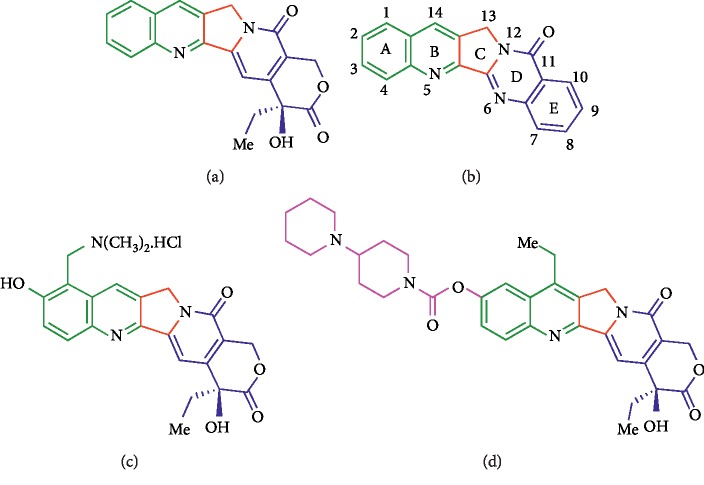
Structures of some CPT-related anticancer agents acting as topoisomerase I inhibitors. (a) Camptothecin. (b) Luotonin A. (c) Topotecan. (d) Irinotecan.

Topoisomerases are present in all living organisms and their function is to relieve torsional tension in supercoiled DNA. This is essential for successful DNA replication, transcription, and reparation [6], and therefore, topoisomerase inhibition or poisoning is an important strategy in cancer chemotherapy [7–10]. Topoisomerase II is the target of a number of anticancer agents in clinical use, including etoposide, amsacrine, and doxorubicin [3, 11]. Other known topoisomerase II targeted anticancer frameworks include quercetin [12] and ellipticine and its derivatives and related heterocycles such as carbazoles [13–15], xanthone-polyamine conjugates [16], polyheterocyclic compounds with a tertiary amino side chain [17], nucleosides [18], and titanocenes [19]. The main drugs that have topoisomerase I as their target are camptothecins (CPTs) (Figure 1) but they suffer from several limitations in spite of being in clinical use. In the first place, the *δ*-lactone moiety in the E-ring of these compounds is highly sensitive to hydrolysis [20]. Furthermore, CPT has very poor solubility in water, which led to the introduction of basic substituents into topotecan and irinotecan, in order to allow the compounds to be formulated as salts. Dual inhibition of topoisomerases I and II by a single agent leads to compounds that are able to target a larger population of cells in comparison with selective inhibitors and increase antitumor activity and has been proposed as a worthwhile goal in the discovery of anticancer agents [21].

Luotonin A, an alkaloid from *Peganum nigelastrum*, can be viewed as a hydrolysis-stable CPT analogue. While both compounds share the same mechanism of action, namely, stabilization of the topoisomerase I−DNA complex, luotonin shows a much lower level of activity. The importance of the development of more potent luotonin A analogues has prompted much synthetic work [22–37], and these efforts have furnished several types of structural variations of the reference alkaloid. Among them, due to limitations in synthetic methodology, the least explored one is the manipulation of the D-ring of luotonin; in this regard, only two examples of D-ring-contracted luotonin analogues have been described [26, 37].

## 2. Materials and Methods

### 2.1. Computational Studies

#### 2.1.1. Crystal Structure Preparation

The starting point for the docking study was the crystal structure of the topoisomerase I-DNA covalent complex bound to topotecan (PDB code 1K4T) [38], the only clinically used camptothecin derivative that has been crystallized in its site of action. A molecule of polyethyleneglycol, an atom of Hg, and the lactone and hydroxyl acid forms of topotecan were manually removed from the crystal. Water molecules were then deleted using the Dockprep tool of Chimera [39], which was also employed to add hydrogen atoms to the enzyme and DNA molecules, repair truncated side chains *via* the Dunbrack rotamer library [40], and assign Gasteiger charges using the AMBER ff12SB force field [41]. From the resulting structure, AutoDock Tools 1.5.6 was used to generate the input file for docking [42], and the docking site for this study was defined as a box with dimensions 15 × 15 × 20 Å. The centroid of this box was calculated using the coordinates of the closed lactone form of topotecan (*x* = 21.377, *y* = −4.068, *z* = 28.192).

#### 2.1.2. Ligand Preparation

The ligands were prepared by *ab initio* energy minimization with Spartan'10 (3-21G level). Hydrogen atoms were then added, and the root of the torsion tree was detected. As in the previous case, the input file for the docking experiments was created with AutoDock Tools 1.5.6.

#### 2.1.3. Docking Studies

We employed AutoDock Vina [43] for the docking studies, using the parameters described above. For the validation of the protocol, we used the closed lactone form of topotecan as the ligand for one of the experiments and compared its most stable docking pose with the crystal structure, obtaining a RMSD value of 0.492 Å.

### 2.2. Synthesis [44]

Melting points of all the compounds were measured by means of open capillary tubes and are uncorrected. ^1^H, ^13^C, and two-dimensional nuclear magnetic resonance spectra were recorded on a Jeol Instruments spectrophotometer (400 and 500 MHz) in CDCl_3_ using TMS as the internal standard. Chemical shifts are given in ppm (*δ*-scale), and the coupling constant values are given in Hertz. Mass spectra were recorded on a Quattro Premier triple quadrupole mass spectrometer equipped with an electrospray ionization source (Z-spray) coupled with an Acquity UPLC system. Elemental analyses were performed on a PerkinElmer 2400 Series II elemental CHNS analyzer.

#### 2.2.1. 1,2,3,4-Tetrahydroisoquinoline-3-carboxylic Acid (2)

The procedure was adapted from the literature [45]. A solution of (±)-phenylalanine (**1**) (2.0 g, 12.12 mmol) in HBr (10 mL) was heated to 40°C. Formaldehyde (1.8 mL, 48.86 mmol) was then added dropwise. The reaction mixture was further heated to 70–80°C for 3 h, during which a white precipitate formed. The reaction mixture was cooled to room temperature, and the precipitate was filtered under vacuum and washed with cold ethanol. The white solid product thus obtained was left under vacuum to dry. Yield = 90% (1.79 g) and melting point = 295–300°C (decomposes).

#### 2.2.2. (±)-Methyl-1,2,3,4-tetrahydroisoquinoline-3-carboxylate (3)

To a stirred, ice-cold suspension of (±)-1,2,3,4-tetrahydroisoquinoline-3-carboxylic acid (1 g, 0.006 mol) in methanol (50 mL), thionyl chloride (2.02 g, 0.018 mol) was added dropwise over 10 minutes, and then, the reaction mixture was heated at reflux for 5 hours and then stirred at ambient temperature overnight; methanol was evaporated in vacuo to obtain a colourless solid. The vacuum-dried (±)-methyl-1,2,3,4-tetrahydroisoquinoline-3-carboxylate hydrochloride was washed with dry diethyl ether, filtered, and dried in vacuo. The solid thus obtained was used as such in the next step. Yield = 96%; melting point = 259–263°C.

#### 2.2.3. (±)-Methyl 2-(propargyl)-1,2,3,4-tetrahydroisoquinoline-3-carboxylate (4)

To a suspension of **3** (0.5 g, 0.002 mol), acetone (10 mL), and K_2_CO_3_ (1.215 g, 0.008 mol) in water (2 mL), which was cooled externally with an ice bath, propargyl bromide (0.393 g, 0.003 mol) in acetone (5 mL) was added dropwise. The reaction mixture was stirred at room temperature for 12 h. The precipitate collected after the removal of the solvent was extracted with ethyl acetate (3 × 50 mL) and dried. Evaporation of ethyl acetate under reduced pressure afforded **4** as a viscous liquid. Yield (quantitative) = 0.500 g, IR (KBr) *ν*_max_ 1010, 1195, 1444, 1736 cm^−1^; ^1^H NMR (400 MHz, CDCl_3_): *δ*_Η_ 2.38 (t, 1H, *J* = 2.92 Hz), 3.25 (t, 2H, *J* = 5.88 Hz), 3.70 (dd, 1H, *J* = 16.84, 2.2 Hz), 3.79 (s, 3H), 3.85 (dd, 1H, *J* = 16.12, 2.2 Hz), 3.97 (t, 1H, *J* = 5.88 Hz), 4.12 (s, 2H), 7.11–7.25 (m, 4H). ^13^C NMR (100 MHz, CDCl_3_) *δ*_C_ 32.35, 44.10, 51.78, 52.07, 59.09, 73.75, 79.11, 126.37, 126.57, 126.60, 128.58, 132.00, 133.93, 173.01.

#### 2.2.4. (±)-[2-(Propargyl)-1,2,3,4-tetrahydroisoquinolin-3-yl]methanol (5)

To a solution of NaBH_4_ (0.5 g, 0.002 mol) in absolute ethanol (15 mL), a solution of **4** (0.332 g, 0.008 mol) in ethanol (5 mL) was added dropwise, and the resulting mixture was refluxed for 15 min. After reaction completion as evident from TLC, ethanol was evaporated in a vacuum. The resulting precipitate was then extracted with ethyl acetate (3 × 50 mL), and product **5** was obtained as a white solid. Yield = 95% (0.415 g), IR (KBr) *ν*_max_ 1088, 1441, 3271 cm^−1^; ^1^H NMR (400 MHz, CDCl_3_): *δ*_Η_ 2.19 (t, 1H, *J* = 2.2 Hz), 2.76–2.78 (m, 2H), 2.98–3.04 (m, 1H), 3.39 (dd, 1H, *J* = 16.88, 2.2 Hz), 3.55 (dd, 1H, *J* = 11.0, 5.12 Hz), 3.62 (dd, 1H, *J* = 16.84, 2.2 Hz), 3.71 (dd, 1H, *J* = 11.0, 4.4 Hz), 3.82 (d, 1H, *J* = 15.26 Hz), 3.91 (d, 1H, *J* = 15.26 Hz), 6.97–7.10 (m, 4H). ^13^C NMR (100 MHz, CDCl_3_): *δ*_C_ 29.38, 42.38, 52.32, 57.01, 62.85, 73.31, 79.06, 125.89, 126.35, 126.46, 128.46, 133.56, 133.89.

#### 2.2.5. (±)-2-(Propargyl)-1,2,3,4-tetrahydroisoquinoline-3-carbaldehyde (6)

To a solution of (COCl)_2_ (0.53 mL, 6.20 mmol) in dry CH_2_Cl_2_ (15 mL) at −50°C, DMSO (0.88 mL, 12.40 mmol) in dry CH_2_Cl_2_ (4 mL) was added dropwise over 30 min. After stirring for 15 min, a solution of **5** (500 mg, 2.48 mmol) in dry CH_2_Cl_2_ (10 mL) was added dropwise over 45 min. Then, the reaction mixture was stirred at −50°C for 16 h. Then, Et_3_N (1.8 mL) was added dropwise over 30 min, and the reaction mixture was stirred for a further 15 min. The organic layer was washed with water (3 × 50 mL) and dried (MgSO_4_). The solvent was removed under vacuum to give **6** as yellow oil. Yield = 94% (0.465 g), IR (KBr) *ν*_max_ 1024, 1264, 1499, 1756 cm^−1^; ^1^H NMR (400 MHz, CDCl_3_): *δ*_Η_ 2.23–2.24 (m, 1H), 2.88–3.04 (m, 2H), 3.50–3.64 (m, 3H), 3.92 (d, 1H, *J* = 20.0 Hz), 3.97 (d, 1H, *J* = 20.0 Hz), 6.99–7.11 (m, 4H), 9.68 (d, 1H, *J* = 3.0 Hz). ^13^C NMR (100 MHz, CDCl_3_): *δ*_C_ 27.98, 44.34, 52.71, 65.47, 74.70, 79.14, 127.04, 127.16, 127.34, 129.09, 132.01, 134.14, 202.75.

#### 2.2.6. General Procedure for the Synthesis of (±)-5b,6,11,13-Tetrahydrobenzo[6,7]indolizino[1,2*b*]-quinolone Derivatives (8)

To a suspension of 4 Å molecular sieves (2 g) in dry CH_2_Cl_2_ under an inert gas atmosphere, a solution of **6** (500 mg, 2.5 mmol) in CH_2_Cl_2_ (15 mL) and an appropriate amount of arylamine (2.5 mmol) in CH_2_Cl_2_ (10 mL) was added. The reaction mixture, after being stirred at room temperature for 12 h, was filtered under an inert gas atmosphere through celite with dry CH_2_Cl_2_ as the eluent. The collected filtrate was concentrated to 20 mL, and at −78°C, BF_3_·O(C_2_H_5_)_2_ (0.110 mL, 3.75 mmol) was added. Then, the reaction mixture was allowed to warm to room temperature and was stirred further for 2 days. The reaction mixture was extracted with CH_2_Cl_2_ (3 × 30 mL), and the obtained residue was purified by flash column chromatography.

#### 2.2.7. (±)-5b,6,11,13-Tetrahydrobenzo[6,7]indolizino[1,2-*b*]quinoline (8a)

Pale yellow viscous liquid, 42% (0.285 g); IR (KBr) *ν*_max_ 1227, 1460, 1507, 1621 cm^−1^; ^1^H NMR (500 MHz, CDCl_3_): *δ*_Η_ 3.18 (dd, 1H, *J* = 16.0, 11.0 Hz, H-6), 3.65 (dd, 1H, *J* = 16.0, 4.0 Hz, H-6), 3.86–3.92 (m, 2H, H-13 and H-5b), 3.95 (d, 1H, *J* = 15.0 Hz, H-11), 4.26 (d, 1H, *J* = 14.5 Hz, H-11), 4.48 (d, 1H, *J* = 13.0 Hz, H-13), 6.90 (d, 1H, *J* = 7.5 Hz, ArH), 7.04–7.12 (m, 3H, ArH), 7.57–7.70 (m, 2H, ArH), 7.97 (d, 1H, *J* = 8.0 Hz, ArH), 8.14 (d, 1H, *J* = 9.0 Hz, ArH), 8.34 (s, 1H, ArH). ^13^C NMR (125 MHz, CDCl_3_): *δ*_C_ 32.84, 52.76, 56.06, 63.52, 120.80, 123.30, 125.32, 125.87, 126.16, 126.35, 127.00, 128.29, 128.51, 128.86, 129.21, 130.27, 133.10, 143.90, 162.35. LC/MS (ESI): 273 (M^+^). Values calculated for C_19_H_16_N_2_: C, 83.79; H, 5.92; N, 10.29. Found: C, 83.92; H, 5.71; N, 10.14%.

#### 2.2.8. (±)-2-Methoxy-5b,6,11,13-tetrahydrobenzo[6,7]indolizino[1,2-*b*]quinoline (8b)

Colourless solid, 49% (0.370 g), mp 157–159°C; IR (KBr) *ν*_max_ 1227, 1459, 1512, 1619 cm^−1^; ^1^H NMR (500 MHz, CDCl_3_): *δ*_Η_ 3.14 (dd, 1H, *J* = 15.5, 11.0 Hz, H-6), 3.61 (dd, 1H, *J* = 16.0, 4.0 Hz, H-6), 3.82–3.87 (m, 2H, H-13 and H-5b), 3.91 (s, 3H, OCH_3_), 3.97 (d, 1H, *J* = 14.5 Hz, H-11), 4.24 (d, 1H, *J* = 14.5 Hz, H-11), 4.45 (d, 1H, *J* = 12.5 Hz, H-13), 7.06–7.21 (m, 4H, ArH), 7.26–7.28 (m, 1H, ArH), 7.32 (dd, 1H, *J* = 9.5, 2.5 Hz, ArH), 7.86 (s, 1H, ArH), 8.01 (d, 1H, *J* = 9.5 Hz, ArH). ^13^C NMR (125 MHz, CDCl_3_): *δ*_C_ 32.59, 54.71, 55.38, 55.86, 63.83, 105.90, 120.86, 125.80, 126.37, 126.63, 127.65, 128.53, 129.62, 130.17, 131.98, 133.94, 134.79, 143.46, 157.39, 162.59. LC/MS (ESI): 303 (M^+^). Values calculated for C_20_H_18_N_2_O: C, 79.44; H, 6.00; N, 9.26. Found: C, 79.61; H, 5.82; N, 9.12%.

#### 2.2.9. (±)-3-Methoxy-5b,6,11,13-tetrahydrobenzo[6,7]indolizino[1,2-*b*]quinoline (8c)

Pale yellow viscous liquid, 45% (0.340 g); IR (KBr) *ν*_max_ 1231, 1463, 1503, 1614 cm^−1^; ^1^H NMR (500 MHz, CDCl_3_): *δ*_Η_ 3.17 (dd, 1H, *J* = 16.0, 11.0 Hz, H-6), 3.65 (dd, 1H, *J* = 16.0, 4.0 Hz, H-6), 3.85–3.89 (m, 2H, H-13 and H-5b), 3.92 (s, 3H, OCH_3_), 3.99 (d, 1H, *J* = 15.0 Hz, H-11), 4.27 (d, 1H, *J* = 14.5 Hz, H-11), 4.46 (d, 1H, *J* = 13.0 Hz, H-13), 7.06–7.31 (m, 5H, ArH), 7.35 (d, 1H, *J* = 9.0 Hz, ArH), 7.88 (s, 1H, ArH), 8.02 (d, 1H, *J* = 9.0 Hz, ArH). ^13^C NMR (125 MHz, CDCl_3_): *δ*_C_ 32.20, 54.67, 55.41, 55.89, 63.80, 105.63, 116.10, 123.72, 125.33, 126.71, 127.60, 128.61, 129.60, 130.12, 131.75, 133.90, 134.84, 143.82, 147.02, 162.50. LC/MS (ESI): 303 (M^+^). Values calculated for C_20_H_18_N_2_O: C, 79.44; H, 6.00; N, 9.26. Found: C, 79.67; H, 6.13; N, 9.35%.

#### 2.2.10. (±)-4-Methoxy-5b,6,11,13-tetrahydrobenzo[6,7]indolizino[1,2-*b*]quinoline (8d)

Yellow solid, 47% (0.355 g), mp 165–167°C; IR (KBr) *ν*_max_ 1224, 1456, 1501, 1618 cm^−1^; ^1^H NMR (500 MHz, CDCl_3_): *δ*_Η_ 3.18 (dd, 1H, *J* = 16.0, 11.5 Hz, H-6), 3.72 (dd, 1H, *J* = 16.0, 4.0 Hz, H-6), 3.83–3.87 (m, 2H, H-13 and H-5b), 3.99 (d, 1H, *J* = 14.0 Hz, H-11), 4.09 (s, 3H, OCH_3_), 4.25 (d, 1H, *J* = 14.5 Hz, H-11), 4.47 (d, 1H, *J* = 13.0 Hz, H-13), 7.04 (d, 1H, *J* = 9.0 Hz, ArH), 7.12–7.26 (m, 4H, ArH), 7.36 (d, 1H, *J* = 9.0 Hz, ArH), 7.42 (d, 1H, *J* = 8.5 Hz, ArH), 7.95 (s, 1H, ArH). ^13^C NMR (125 MHz, CDCl_3_): *δ*_C_ 32.78, 52.86, 54.81, 55.87, 64.31, 107.46, 119.85, 125.88, 126.18, 126.48, 126.72, 128.80, 128.90, 129.74, 132.38, 134.22, 134.84, 139.42, 155.27, 164.12. LC/MS (ESI): 303 (M^+^). Values calculated for C_20_H_18_N_2_O: C, 79.44; H, 6.00; N, 9.26. Found: C, 79.62; H, 6.19; N, 9.10%.

#### 2.2.11. (±)-2-Methyl-5b,6,11,13-tetrahydrobenzo[6,7]indolizino[1,2-*b*]quinoline (8e)

Pale yellow viscous liquid, 44% (0.315 g); IR (KBr) *ν*_max_ 1235, 1450, 1510, 1624 cm^−1^; ^1^H NMR (500 MHz, CDCl_3_): *δ*_Η_ 2.52 (s, 3H, CH_3_), 3.15 (dd, 1H, *J* = 15.5, 11.0 Hz, H-6), 3.63 (dd, 1H, *J* = 15.5, 4.0 Hz, H-6), 3.83–3.88 (m, 2H, H-13 and H-5b), 3.98 (d, 1H, *J* = 14.5 Hz, H-11), 4.25 (d, 1H, *J* = 14.5 Hz, H-11), 4.46 (d, 1H, *J* = 13.5 Hz, H-13), 6.59 (d, 1H, *J* = 8.0 Hz, ArH), 6.96 (d, 1H, *J* = 8.0 Hz, ArH), 7.15–7.22 (m, 2H, ArH), 7.50–7.57 (m, 2H, ArH), 7.88 (s, 1H, ArH), 8.01 (d, 1H, *J* = 8.5 Hz, ArH). ^13^C NMR (125 MHz, CDCl_3_): *δ*_C_ 21.48, 32.64, 54.84, 55.42, 64.04, 115.23, 119.93, 125.91, 126.49, 126.74, 126.87, 127.69, 128.24, 128.64, 129.58, 131.04, 131.70, 134.86, 143.78, 164.14. LC/MS (ESI): 287 (M^+^). Analysis calculated for C_20_H_18_N_2_: C, 83.88; H, 6.34; N, 9.78. Found: C, 83.75; H, 6.45; N, 9.97%.

#### 2.2.12. (±)-3-Methyl-5b,6,11,13-tetrahydrobenzo[6,7]indolizino[1,2-*b*]quinoline (8f)

Pale yellow viscous liquid, 43% (0.307 g); IR (KBr) *ν*_max_ 1221, 1457, 1508, 1623 cm^−1^; ^1^H NMR (500 MHz, CDCl_3_): *δ*_Η_ 2.63 (s, 1H, CH_3_), 3.14 (dd, 1H, *J* = 15.0, 11.0 Hz, H-6), 3.68 (dd, 1H, *J* = 15.0, 4.0 Hz, H-6), 3.85 (d, 1H, *J* = 12.5 Hz, H-13), 3.93–3.96 (m, 1H, H-5b), 4.06 (d, 1H, *J* = 15.0 Hz, H-11), 4.31 (d, 1H, *J* = 15.0 Hz, H-11), 4.47 (d, 1H, *J* = 12.5 Hz, H-13), 7.08–7.35 (m, 5H, ArH), 7.39 (d, 1H, *J* = 8.5 Hz, ArH), 7.90 (s, 1H, ArH), and 8.01 (d, 1H, *J* = 8.0 Hz, ArH). ^13^C NMR (125 MHz, CDCl_3_): *δ*_C_ 20.05, 32.65, 54.80, 55.93, 64.20, 125.51, 125.78, 125.84, 126.46, 126.70, 127.49, 128.69, 128.98, 129.62, 131.17, 134.39, 134.90, 137.10, 146.48, 163.65. LC/MS (ESI): 287 (M^+^). Values calculated for C_20_H_18_N_2_: C, 83.88; H, 6.34; N, 9.78. Found: C, 83.67; H, 6.20; N, 9.65%.

#### 2.2.13. (±)-4-Methyl-5b,6,11,13-tetrahydrobenzo[6,7]indolizino[1,2-*b*]quinoline (8g)

Colourless solid, 41% (0.294 g), mp 142–144°C; IR (KBr) *ν*_max_ 1230, 1466, 1514, 1622 cm^−1^; ^1^H NMR (500 MHz, CDCl_3_): *δ*_Η_ 2.87 (s, 1H, CH_3_), 3.18 (dd, 1H, *J* = 15.5, 11.0 Hz, H-6), 3.67 (dd, 1H, *J* = 15.5, 3.5 Hz, H-6), 3.87 (d, 1H, *J* = 13.0 Hz, H-13), 3.91–3.93 (m, 1H, H-5b), 4.02 (d, 1H, *J* = 15.0 Hz, H-11), 4.28 (d, 1H, *J* = 15.0 Hz, H-11), 4.48 (d, 1H, *J* = 12.5 Hz, H-13), 7.16–7.29 (m, 3H, ArH), 7.32 (d, 1H, *J* = 8.0 Hz, ArH), 7.40–7.43 (m, 1H, ArH), 7.55 (d, 1H, *J* = 7.0 Hz, ArH), 7.65 (d, 1H, *J* = 8.0 Hz, ArH), 7.93 (s, 1H, ArH). ^13^C NMR (125 MHz, CDCl_3_): *δ*_C_ 19.18, 32.68, 54.83, 55.85, 64.26, 125.59, 125.76, 125.80, 126.41, 126.71, 127.44, 128.76, 128.96, 129.65, 131.13, 134.20, 134.93, 136.96, 146.70, 163.73. LC/MS (ESI): 287 (M^+^). Analysis calculated for C_20_H_18_N_2_: C, 83.88; H, 6.34; N, 9.78. Found: C, 83.79; H, 6.49; N, 9.65%.

#### 2.2.14. (±)-2-Chloro-5b,6,11,13-tetrahydrobenzo[6,7]indolizino[1,2-*b*]quinoline (8h)

Pale yellow viscous liquid, 40% (0.308 g); IR (KBr) *ν*_max_ 1226, 1451, 1522, 1620 cm^−1^; ^1^H NMR (500 MHz, CDCl_3_): *δ*_Η_ 3.19 (dd, 1H, *J* = 15.5, 10.5 Hz, H-6), 3.63 (dd, 1H, *J* = 15.5, 4.0 Hz, H-6), 3.85–3.94 (m, 2H, H-13 and H-5b), 3.99 (d, 1H, *J* = 14.5 Hz, H-11), 4.27 (d, 1H, *J* = 15.0 Hz, H-11), 4.49 (d, 1H, *J* = 12.5 Hz, H-13), 7.05–7.23 (m, 4H, ArH), 7.27–7.31 (m, 1H, ArH), 7.33–7.39 (m, 1H, ArH), 7.90 (s, 1H, ArH), 8.04 (d, 1H, *J* = 9.0 Hz, ArH). ^13^C NMR (125 MHz, CDCl_3_): *δ*_C_ 31.90, 54.39, 57.87, 63.54, 120.14, 121.09, 122.57, 123.29, 125.87, 128.08, 128.51, 128.96, 130.17, 130.38, 130.92, 132.94, 134.21, 143.52, 163.02. LC/MS (ESI): 308 (M^+^). Analysis calculated for C_19_H_15_ClN_2_: C, 74.38; H, 4.93; N, 9.13. Found: C, 74.51; H, 4.71; N, 9.29%.

#### 2.2.15. (±)-4-Chloro-5b,6,11,13-tetrahydrobenzo[6,7]indolizino[1,2-*b*]quinoline (8i)

Pale yellow viscous liquid, 38% (0.290 g); IR (KBr) *ν*_max_ 1230, 1467, 1504, 1626 cm^−1^; ^1^H NMR (500 MHz, CDCl_3_): *δ*_Η_ 3.14 (dd, 1H, *J* = 15.0, 11.0 Hz, H-6), 3.65 (dd, 1H, *J* = 15.5, 3.5 Hz, H-6), 3.90 (d, 1H, *J* = 12.5 Hz, H-13), 3.93–3.96 (m, 1H, H-5b), 4.06 (d, 1H, *J* = 15.0 Hz, H-11), 4.30 (d, 1H, *J* = 15.0 Hz, H-11), 4.49 (d, 1H, *J* = 12.5 Hz, H-13), 7.19–7.37 (m, 4H, ArH), 7.42–7.47 (m, 1H, ArH), 7.58 (d, 1H, *J* = 8.0 Hz, ArH), 7.69 (d, 1H, *J* = 8.0 Hz, ArH), 7.98 (s, 1H, ArH). ^13^C NMR (125 MHz, CDCl_3_): *δ*_C_ 32.36, 54.80, 55.61, 63.94, 125.54, 125.79, 125.93, 126.47, 126.72, 127.56, 128.74, 128.98, 129.62, 131.20, 134.36, 134.94, 136.98, 138.72, 163.62. LC/MS (ESI): 308 (M^+^). Analysis calculated for C_19_H_15_ClN_2_: C, 74.38; H, 4.93; N, 9.13. Found: C, 74.22; H, 4.80; N, 9.27%.

### 2.3. Biology

#### 2.3.1. Stock Preparation

For the cytotoxicity assays, stock solutions of the compounds (**8a–i**) were prepared in DMSO, and during the experiments involving cell cultures, required amounts of the compounds were directly mixed with the culture media such that the final concentration of DMSO was below 0.5%.

#### 2.3.2. Cell Cultures

Four different cell lines, namely, MCF7 (human breast cancer), HCT116 (human colon cancer), NCI-H460 (human non-small-cell lung cancer), and JURKAT (human T-cell lymphoma) cell lines, were obtained from NCCS, Pune, India. All the cell lines were cultured in DMEM (Dulbecco's modified Eagle's medium), except JURKAT, which was cultured in RPMI (Roswell Park Memorial Institute) media supplemented with 10% FBS (fetal bovine serum) (Himedia, India), containing penicillin (100 I.U/mL) and streptomycin (100 *μ*g/mL) at 37°C in a humidified atmosphere of 5% CO_2_ in air. For compound testing, the adherent cells were trypsinized, counted, and seeded in 96-well plates for viability studies at a density of 20 × 10^3^ cells per well, where they were left to adhere overnight before the experiments with our compounds. The compounds under assay were not added until the wells were observed to show at least 80% confluence [46].

#### 2.3.3. Cytotoxicity Assay

The cytotoxic behaviour of compounds **8a–i** on adherent cells was determined by the MTT (i.e., 3-(4,5-dimethylthiazol-2-yl)-2,5-diphenyltetrazolium bromide) reduction assay. Prior to treatment (12–14 h), the cells were seeded in a 96-well plate at a density of 20 × 10^3^ cells per well. The cell media contained in the wells were discarded, and the cells were treated with compounds **8a–i** at 125 *μ*M concentration. This concentration was chosen because it guaranteed all compounds to show at least some cytotoxicity to allow comparison. These treatments were maintained for 24 or 48 h. When the experiments ended, the cells were washed and promptly assayed for viability using MTT. The absorbances of the wells containing treated and untreated cells were measured on a microplate reader at 570 nm wavelength. JURKAT cells were seeded as above, along with different concentrations of compounds under assay in 96-well plates at a density of 20000 cells per well and incubated for 24 or 48 h at 37°C in a CO_2_ incubator. The MTT assay was performed as per the instructions from the manufacturer, and the absorbance of the wells with treated and untreated cells was measured on a microplate reader at 570 nm wavelength. The results were represented as the percentage of viability = {[A570 (treated cells) − background]/[A570 (untreated cells) − background]} × 100. Each treatment was performed at least in triplicate. CPT at 40 *μ*M concentration was used as a positive control [47].

#### 2.3.4. Topoisomerase I Mediated DNA Relaxation Assay

**Figure 2:**
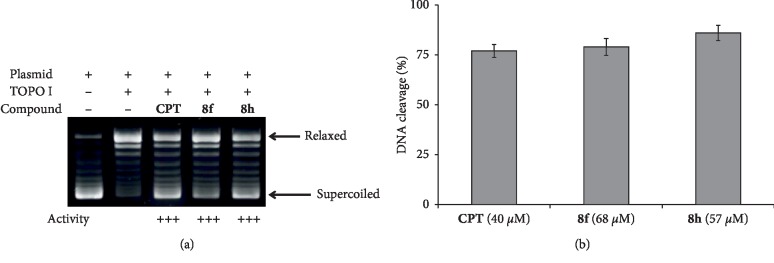
(a) DNA relaxation inhibition assay for compounds **8f** (at 68 *μ*M concentration) and **8h** (at 57 *μ*M concentration) and CPT (of 40 *μ*M), and (b) the corresponding quantitation graph. *∗* corresponds to the significant (*p* < 0.05) values, and *∗∗* corresponds to the highly significant (*p* < 0.01) values.

The inhibitory effect of compounds **8f** and **8h** on the activity of human topoisomerase I was assayed by means of a DNA relaxation assay. To this end, a final reaction volume of 30 *μ*L containing the reaction buffer, the pGEM-5Zf(+) plasmid (Promega) in its native supercoiled form (200 ng), and the compounds under assay (the reference drug **CPT** and compounds **8f** and **8h**) were diluted in 1 mL DMSO to achieve their IC_50_ concentrations, i.e., 40 *μ*M, 68 *μ*M, and 57 *μ*M, respectively, and 1 unit of human topoisomerase I (Inspiralis) was incubated at 37°C for 30 min. The reaction corresponding to the native supercoiled form was carried out from a mixture containing the reaction buffer and plasmid (lane 1, Figure 2(a)) and that corresponding to the fully relaxed form was carried out from the reaction buffer, plasmid, and human topoisomerase I (lane 2, Figure 2(a)). For the remaining three experiments, the compounds assayed were also included (lanes 3–5). Following incubation, the reactions were stopped by the addition of a mixture of the buffer employed for the subsequent electrophoresis and 20% SDS. The reaction products were loaded onto 1% agarose gel (GLPL), and an electrophoresis experiment was run at 4°C for 3 h at 100 V in Tris-borate-EDTA buffer. The gels were stained with ethidium bromide from Sigma, using the manufacturer's protocol. Then, they were photographed under 254 nm UV light using a gel doc (UVitech) system, and the bands were analyzed using the inbuilt software. The inhibitory activities of the compounds under assay were calculated using the ratio of intensities of supercoiled to unwinded bands and normalized to the activity of CPT.

#### 2.3.5. Topoisomerase II Decatenation Assay

Reactions contained 0.15 *μ*g kinetoplast DNA (kDNA), the fresh assay buffer, 2 units of human topoisomerase II*α*, and compounds **8f** (68 *μ*M), **8h** (57 *μ*M), and **CPT** (40 *μ*M) in a 20 *μ*L final volume. These reaction mixtures were incubated for 45 min at 37°C. The reactions were terminated with 4 *μ*L of 5x stop loading buffer (5% Sarkosyl, 0.12% bromophenol blue, and 25% glycerol) and were loaded directly onto 1% agarose gel in TAE buffer. The gel was stained with ethidium bromide and detained in water before being photographed under UV light.

#### 2.3.6. Statistical Analysis

All the in vitro results given here were calculated as mean ± SD of at least three replicate analyses. The statistical analysis of the results was based on Student's *t*-test. Statistical significance was set at *p* < 0.05 (represented by ^*∗*^), and *p* < 0.01 (represented by ^*∗∗*^) was considered a highly significant value. These calculations were performed with the aid of Graphpad Prism software (version 6).

## 3. Results and Discussion

### 3.1. Library Design

**Figure 3:**
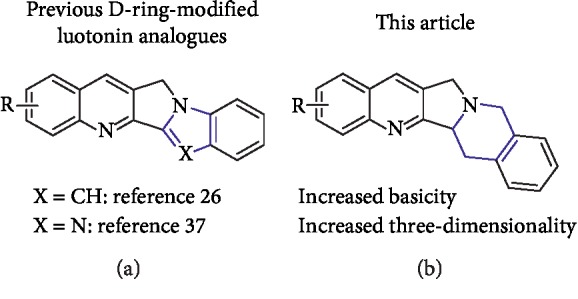
Comparison between previously studied D-ring-modified luotonin analogues and the compounds studied here.

The aforementioned limitations in existing topoisomerase I inhibitors prompted us to study the synthesis and biological evaluation of D-ring-modified luotonin analogues. Because the low solubility of CPT derivatives can be ascribed to the high stability of their crystalline lattices in the solid state, which is due to strong intermolecular interactions between their planar polycyclic frameworks, we decided to modify the polycyclic system by replacing the D-ring pyrimidinone unit with a piperidine unit, as shown in Figure 3. This structural modification leads to increased three-dimensionality and hence is expected to be associated with the disruption of crystal packing via decreased planarity, which is a well-established, if relatively unexplored, strategy for increasing the solubility of organic compounds [48]. Furthermore, the proposed modification has the advantage of allowing the easy formation of salts at the strongly basic N-12 cyclic tertiary amine nitrogen.

**Figure 4:**
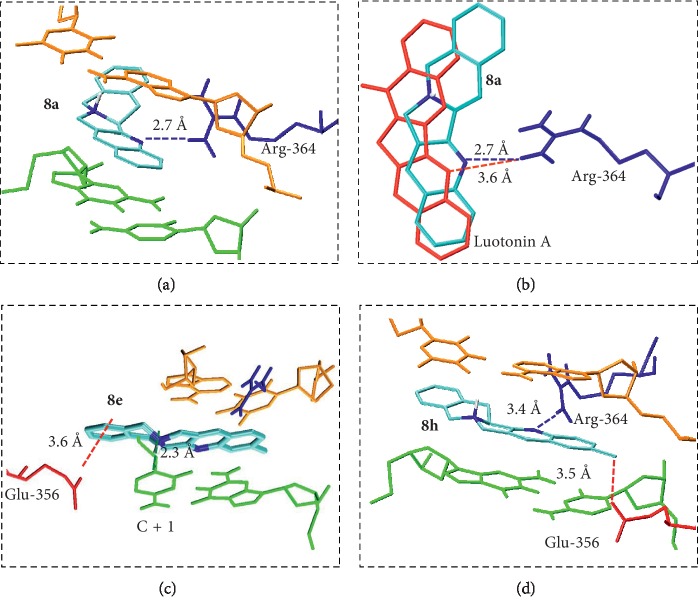
(a) Docking of compound **8a**, the unsubstituted tetrahydrobenzo[6,7]indolizino[1,2-b]quinoline framework, onto the topoisomerase I–DNA complex. (b) Overlay of the docking poses of luotonin A and compound **8a**. (c) Docking of the 2-methyl derivative (compound **8e**). (d) Docking of the 2-chloro derivative (compound **8h**).

In order to address the potential negative effects of diminished planarity on the efficiency of the DNA intercalation process, we carried out docking studies of the unsubstituted tetrahydrobenzo[6,7]indolizino[1,2-b]quinoline framework (compound **8a**) onto the DNA-topoisomerase I covalent complex, which is generated in the course of the enzyme catalytic cycle and is the target of the CPT and luotonin. This study was carried out on the crystal structure of topotecan bound to the same complex, which is available in the Protein Data Bank (PDB 1K4T) [38].  Figure 4 shows the results for the docking of **8a** compared with those of luotonin and shows that in spite of the loss of planarity, **8a** maintains the interaction with the Arg-364 residue, with a shorter binding distance than luotonin, together with *π*−*π* stacking interactions (Figures 4(a) and 4(b)). While the binding free energy was somewhat lower than that of luotonin (−10.2 vs. –11.4 kcal/mol), we hoped to compensate for this difference by the introduction of substituents allowing more efficient binding modes. For instance, the presence of a 2-methyl substituent (compound **8e**) hampers the usual binding but the overall energy is slightly improved (−10.9 kcal/mol) even though the interaction with Arg-364 is lost because two very efficient *π*-cation interactions are now possible (Figure 4(c)). On the other hand, the 2-chloro derivative **8h** retains the binding mode of the parent compound because it allows a halogen bond with Glu-356 and a weak cation-dipole interaction with T-1(C=O), leading again to an improved binding energy (−10.9 kcal/mol) (Figure 4(d)).

### 3.2. Synthesis

**Scheme 1:**
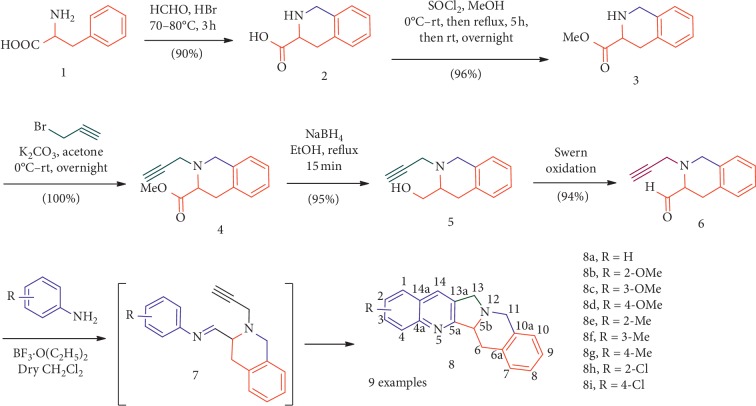
Synthesis of analogues of luotonin A (**8a–i**).

The synthesis of luotonin analogues **8** was scheduled via a Pictet–Spengler/N-propargylation/intramolecular Povarov sequence of reactions. First, the compound 1,2,3,4-tetrahydroisoquinoline-3-carboxylic acid (**2**) was prepared in an excellent yield (90%) from (±)-phenylalanine (**1**) via Pictet–Spengler condensation. The reaction of compound **2** with thionyl chloride afforded the carboxylate derivative (**3**) [45], which upon N-alkylation with propargyl bromide furnished compound **4**. An excellent yield of compound **5** was achieved through sodium borohydride reduction of **4**. Swern oxidation of compound **5** employing DMSO-oxalyl chloride furnished the carbaldehyde **6** in an excellent yield. Finally, the reaction of carbaldehyde **6** with substituted arylamines in the presence of 4 Å molecular sieves afforded the target luotonin analogues **8** in moderate yields through intramolecular Povarov reaction (Scheme1). The synthetic part of this work has been published recently as a preliminary communication [44].

The structural elucidation of compounds **4**–**6** and **8** was performed by NMR studies. A reasonable mechanism for the formation of **8** involves the reaction of aldehyde **6** with arylamine to furnish arylimine **7** and a formal [4 + 2] intramolecular cycloaddition followed by a 1,3-hydrogen shift and air oxidation steps to furnish **8**.

### 3.3. Biological Studies

**Figure 5:**
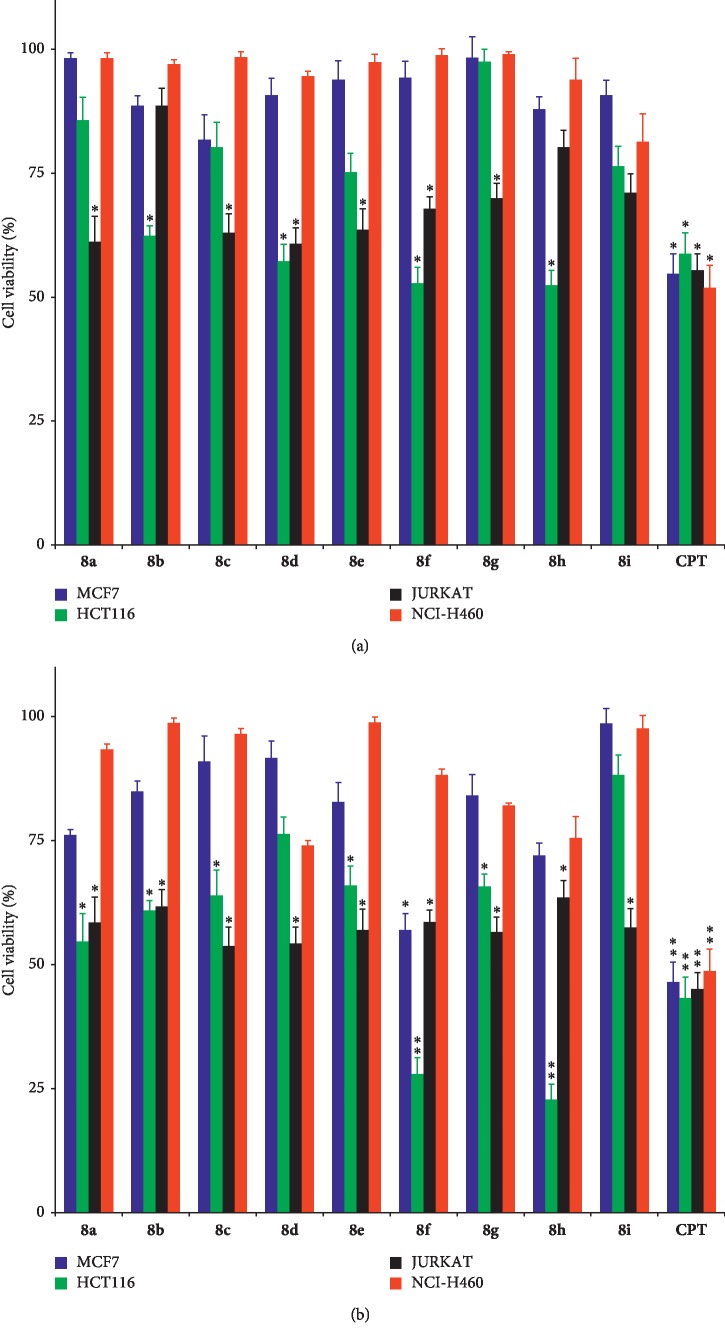
Comparison of the cell viability (%) for MCF7, HCT116, JURKAT, and NCI-H460 cell lines treated with compounds **8a–i** at 125 *μ*M concentration over two different periods of (a) 24 h and (b) 48 h. Here, *∗* represents the significant (*p* < 0.05) values and *∗∗* represents the highly significant (*p* < 0.01) values.

A cytotoxicity study of the luotonin analogues **8a–i** was performed in order to examine their anticancer activity in four human cancer cell lines, namely, MCF7 (breast cancer), HCT116 (colon cancer), JURKAT (T-cell leukemia), and NCI-H460 (non-small-cell lung cancer), with CPT as a positive control. The synthesized compounds were first tested at a concentration of 125 *μ*M for two different incubation times of 24 and 48 h, and the results are shown in Figure 5. In general, the activity order of the tested compounds against four different cancer cells lines is MCF7 < NCI-H460 < JURKAT < HCT116. The loss in cell viability was generally higher with an increased incubation period, i.e., upon moving from 24 to 48 h.

**Figure 6:**
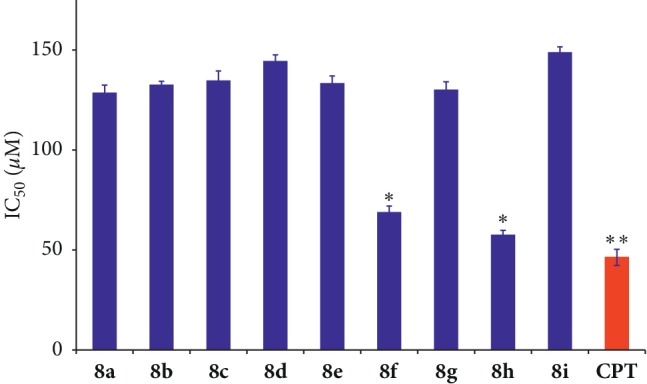
Comparison of the IC_50_ concentration (amount required for 50% cell death) of HCT116 cells following the treatment of compounds **8a–i** over a 48 h period. CPT (40 *μ*M) was used as a positive control. *∗* corresponds to the significant (*p* < 0.05) values and *∗∗* corresponds to the highly significant (*p* < 0.01) values.

Following this initial exploration, all cells were treated with increasing concentrations of the compounds, and the obtained concentration values for 50% cell death (IC_50_) are shown in Table 1 and also in Figure 6 for the specific case of the HCT116 cell line, which showed the highest sensitivity. It is interesting to note that in this cell line, two of the compounds (**8f** and **8h**) were slightly more active than the CPT positive control, which represents a very good level of activity for a luotonin analogue. For this reason, these two compounds were selected for further mechanistic studies. We next investigated the toxicity of compounds **8a–i** on the nontumoral cell line NCM60 (normal human colon mucosal epithelial cell line). For all compounds, no loss in the viability of cells was observed when tested for both time periods of 24 and 48 h in the concentration range of 25–125 *μ*M, thereby indicating the nontoxic nature of our compounds towards nontarget cells. The rationale for the choice of this particular cell line was the fact that it corresponds to colon cells, as HCT116. Furthermore, one of the major dose-limiting toxic effects found for CPT derivatives in clinical use, such as irinotecan, is the frequent appearance of severe diarrhea [49], and thus, the study of the NCM60 cells provides useful toxicological information.

In order to study the mechanism of action of our compounds, their inhibitory activity against purified human topoisomerase I was investigated by means of a relaxation assay that was performed by simultaneous incubation of supercoiled plasmid, pGEM-5Zf (+) DNA, enzyme, and the two more active compounds (**8f** at 68 *μ*M and **8h** at 57 *μ*M). The inhibitory activity of the compounds was compared to that of standard topoisomerase I poison CPT, the same reference used in the cell-based cytotoxicity assays.

An agarose gel showing the separation of DNA topoisomers after incubation of the plasmid in the presence of human topoisomerase I and the inhibitors under assay is shown in Figure 2(a). From the figure, it is clear that the compounds **8f** and **8h** (lanes 4 and 5) showed very good activity that was comparable to that of the CPT control (lane 3). A quantitation graphic is given in Figure 2(b), again showing activity similar to that of CPT for both compounds, which represents an unusually high potency for a luotonin derivative.

**Figure 7:**
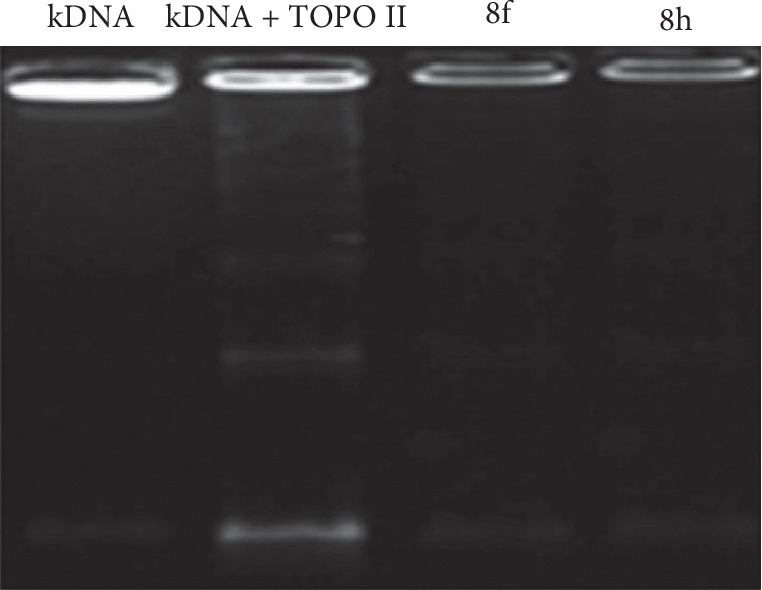
Topoisomerase II inhibition assays showing the effect of **8f** and **8h** (at IC_50_ concentrations) on the decatenation of kDNA by 2U of human topoisomerase II.

As mentioned above, dual inhibition of topoisomerases I and II is an increasingly attractive goal in the design of anticancer agents. Furthermore, batracyclin, a fused quinazoline derivative with some structural resemblance to luotonin, has been characterized as a dual inhibitor of DNA topoisomerases I and II [50]. For these reasons, we also investigated the effect of compounds **8f** and **8h** on the decatenation of kDNA by human topoisomerase II. The appearance of kDNA monomers was monitored in the reaction in order to determine whether an open circular decatenated kDNA form or a closed circular decatenated kDNA form had developed. If Topo II retains its normal function, catenated kDNA would disappear and bands for open circular intermediates and closed circular decatenated kDNA would appear. In contrast, the absence of these bands indicates inhibition of the enzyme. As shown in Figure 7, compounds **8f** and **8h** at their IC_50_ concentrations were found to inhibit Topo II. Therefore, these compounds can be viewed as dual inhibitors of both topoisomerase enzymes. Compounds that target Topo II can be divided into two classes, namely, poisons and catalytic inhibitors [51, 52]. The first group includes most drugs in clinical use and leads to an increase in the levels of Topo II-DNA covalent complexes. A second class of compounds inhibits topo II activity but does not increase the levels of enzyme-DNA complexes. However, the precise mechanism by which our compounds act on their target cannot be presently established.

## 4. Conclusions

A boron trifluoride diethyl etherate-catalyzed intramolecular hetero-Diels–Alder reaction provided convenient access to novel tetrahydrobenzo[6,7]indolizino[1,2-b]quinolone derivatives, designed as nonplanar analogues of the natural topoisomerase I inhibitor luotonin A by means of computational studies. Some of these compounds displayed good cytotoxicity against four different human cancer cell lines (MCF7, HCT116, JURKAT, and NCI-H460). In particular, compounds bearing 3-CH3 (**8f**) and 2-Cl (**8h**) substituents have a good cytotoxic effect on the HCT116 cancer cell line. Compounds **8f** and **8h** were also potent topoisomerase I and II inhibitors. These results indicate that complete planarity is not a strict requirement for topoisomerase inhibition by luotonin-related compounds, paving the way to the design of analogues with improved aqueous solubility.

## Figures and Tables

**Table 1:** Calculated IC_50_ values for the compounds tested (**8a–i**) towards MCF7, HCT116, JURKAT, and NCI-H460 cells for a 48 h period. Standard errors are given within parentheses.

Compounds	IC_50_ (*μ*M; 48 h)			
	MCF7	HCT116	JURKAT	NCI-H460
**8a**	213.56 (±3.45)	128.36 (±4.07)	148.54 (±2.75)	186.43 (±3.15)
**8b**	337.86 (±4.70)	132.34 (±2.04)	261.89 (±3.45)	280.51 (±2.25)
**8c**	335.65 (±2.25)	134.43 (±3.94)	146.78 (±3.15)	172.78 (±1.95)
**8d**	304.12 (±3.25)	144.16 (±3.45)	147.23 (±2.25)	221.89 (±3.85)
**8e**	324.54 (±3.80)	133.12 (±3.95)	158.45 (±2.40)	219.63 (±2.45)
**8f**	98.54 (±2.65)	68.60 (±3.35)	76.46 (±2.45)	84.47 (±2.15)
**8g**	146.54 (±3.75)	129.87 (±2.45)	176.99 (±3.25)	188.65 (±3.45)
**8h**	87.65 (±2.55)	57.35 (±2.45)	69.12 (±2.07)	76.36 (±2.65)
**8i**	140.73 (±3.07)	148.56 (±3.00)	128.45 (±2.15)	176.54 (±1.27)
**CPT**	41.59 (±3.85)	40.27 (±3.05)	41.48 (±3.15)	42.61 (±2.45)

## Data Availability

The data used to support the findings of this study are included within the supplementary information file.

## Supplementary Materials

Supplementary Materials Figure S1: ^1^H NMR spectrum of **4**. Figure S2: ^13^C NMR spectrum of **4**. Figure S3: FT-IR spectrum of **4**. Figure S4: ^1^H NMR spectrum of **5**. Figure S5: ^13^C NMR spectrum of **5**. Figure S6: FT-IR spectrum of **5**. Figure S7: ^1^H NMR spectrum of **6**. Figure S8: ^13^C NMR spectrum of **6**. Figure S9: FT-IR spectrum of **6**. Figure S10: ^1^H NMR spectrum of **8d**. Figure S11: ^13^C NMR spectrum of **8d**. Figure S12: DEPT-135 NMR spectrum of **8d**. Figure S13: H-H COSY spectrum of **8d**. Figure S14: C-H COSY spectrum of **8d**. Figure S15: HMBC spectrum of **8d**. Figure S16: mass spectrum of **8d**. Figure S17: FT-IR spectrum of **8d**. Table S1: experimental details. Table S2: geometric parameters (Å, °).
