# Supplementary material for: D-Ring-Modified Analogues of Luotonin A with Reduced Planarity: Design, Synthesis, and Evaluation of Their Topoisomerase Inhibition-Associated Cytotoxicity
Source: Biomed Res Int. 2019 Nov 13;2019:2514524. doi: 10.1155/2019/2514524 (PMC6877907; doi:10.1155/2019/2514524)
Supplement: Supplementary Materials — Figure S1: 1H NMR spectrum of 4. Figure S2: 13C NMR spectrum of 4. Figure S3: FT-IR spectrum of 4. Figure S4: 1H NMR spectrum of 5. Figure S5: 13C NMR spectrum of 5. Figure S6: FT-IR spectrum of 5. Figure S7: 1H NMR spectrum of 6. Figure S8: 13C NMR spectrum of 6. Figure S9: FT-IR spectrum of 6. Figure S10: 1H NMR spectrum of 8d. Figure S11: 13C NMR spectrum of 8d. Figure S12: DEPT-135 NMR spectrum of 8d. Figure S13: H-H COSY spectrum of 8d. Figure S14: C-H COSY spectrum of 8d. Figure S15: HMBC spectrum of 8d. Figure S16: mass spectrum of 8d. Figure S17: FT-IR spectrum of 8d. Table S1: experimental details. Table S2: geometric parameters (Å, °). [file 2514524.f1.pdf]

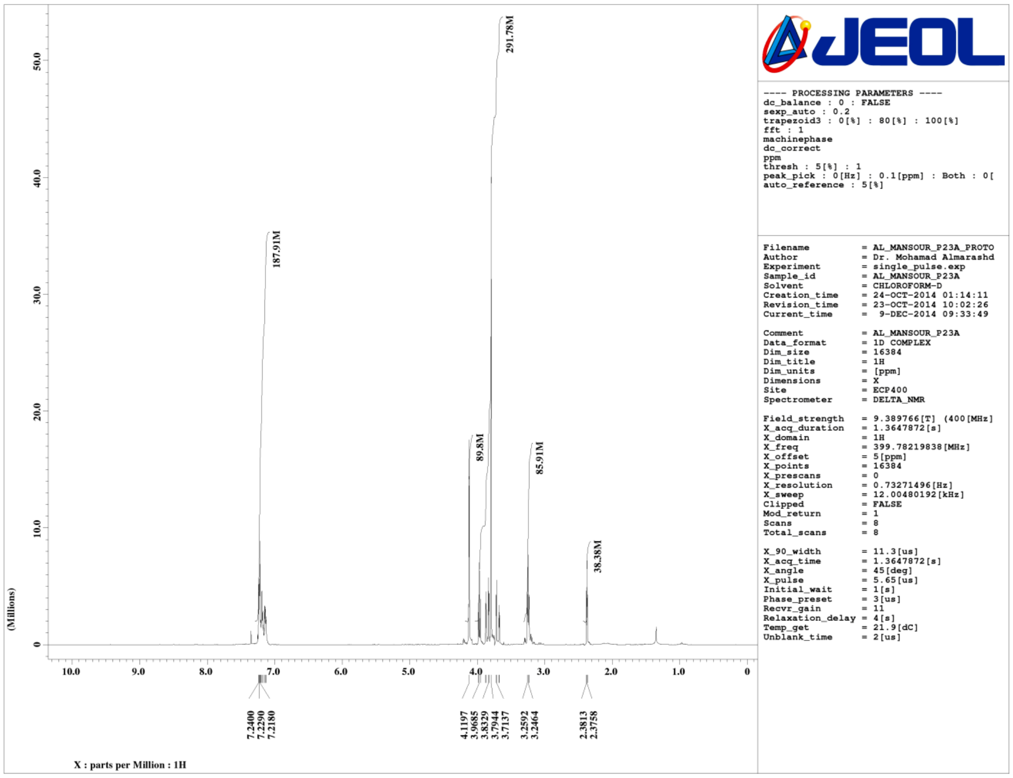

Figure S1 ^{1}H NMR spectrum of **4**

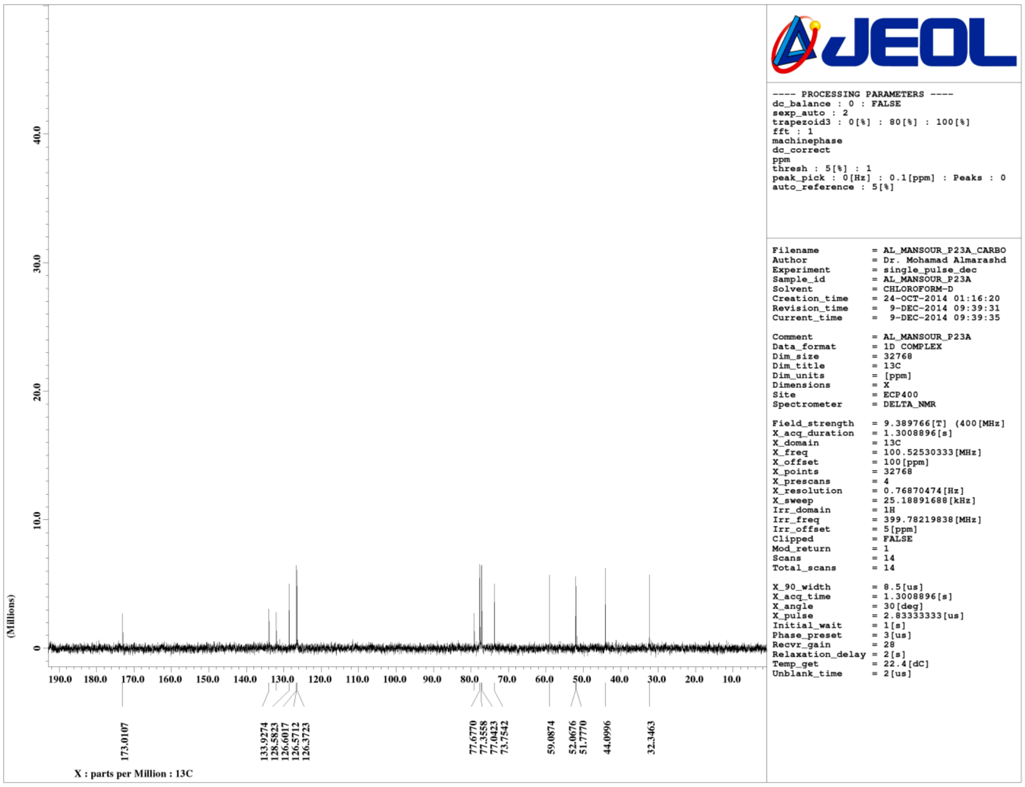

Figure S2 ^{13}C NMR spectrum of **4**

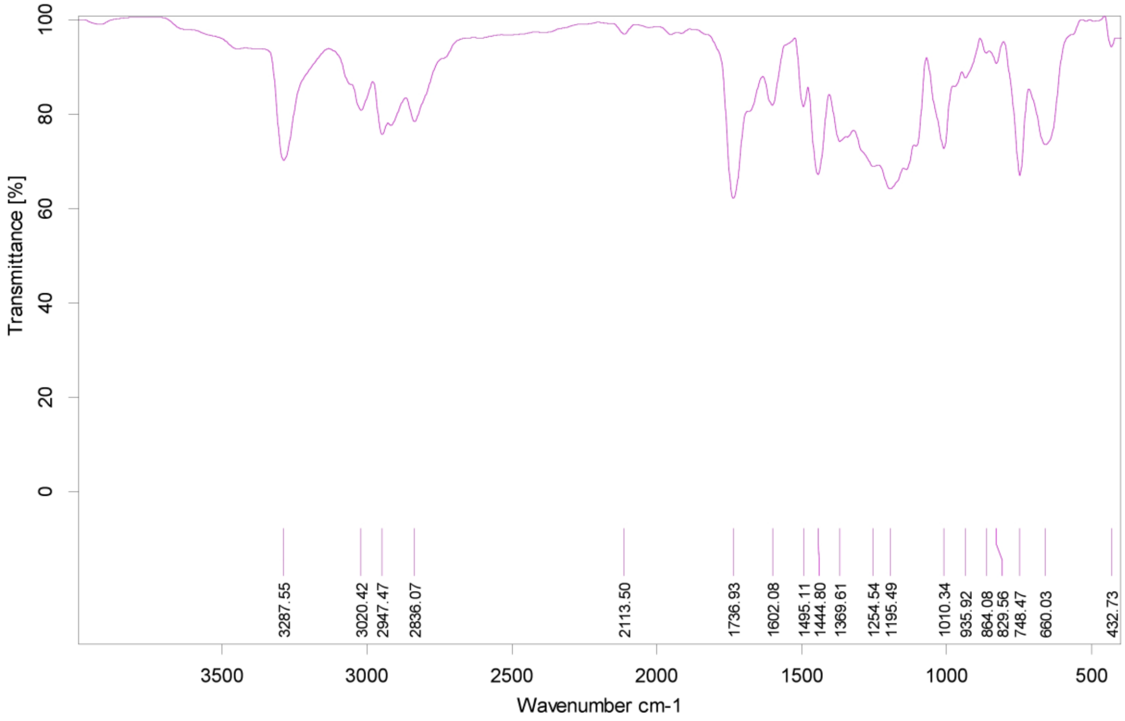

Figure S3 FT-IR spectrum of **4**

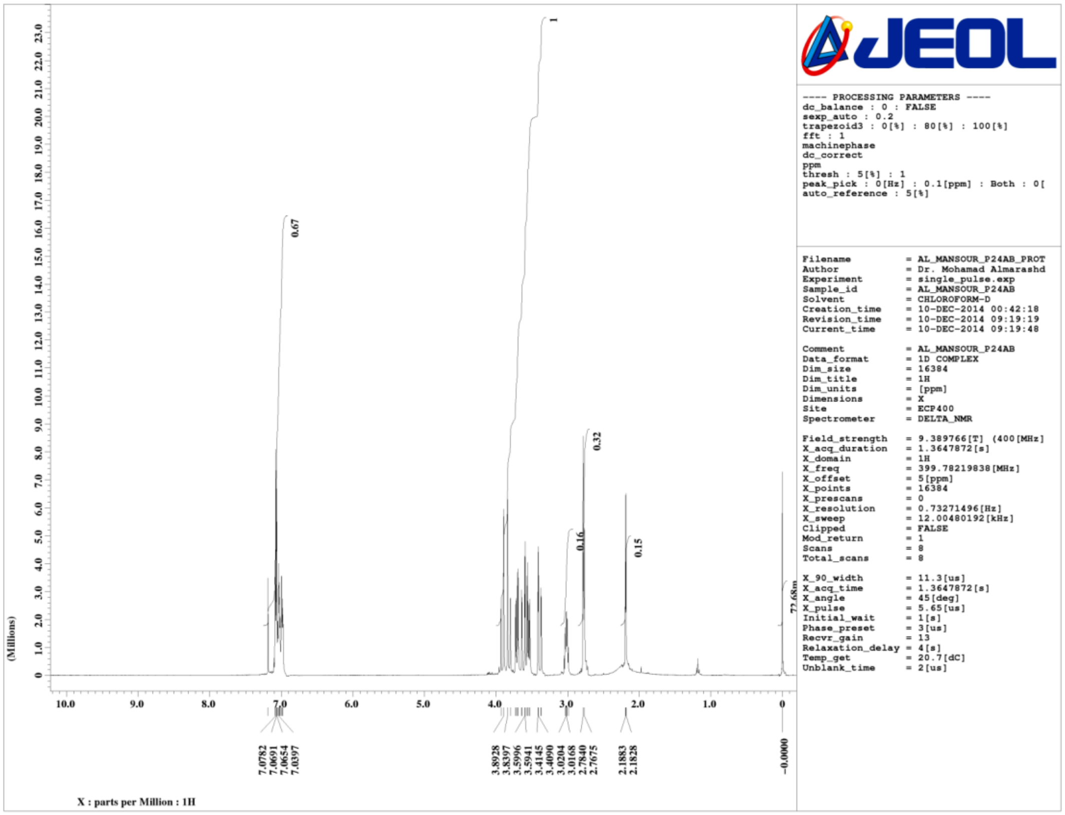

Figure S4 ^{1}H NMR spectrum of **5**

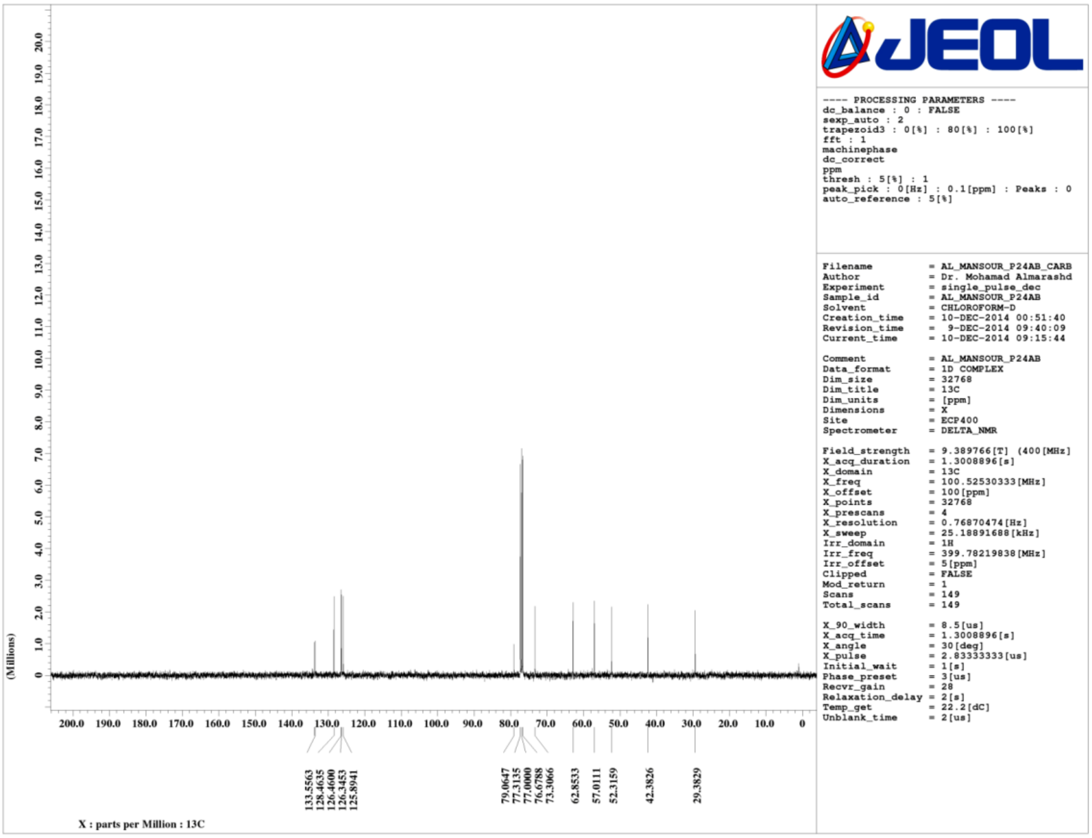

Figure S5 ^{13}C NMR spectrum of **5**

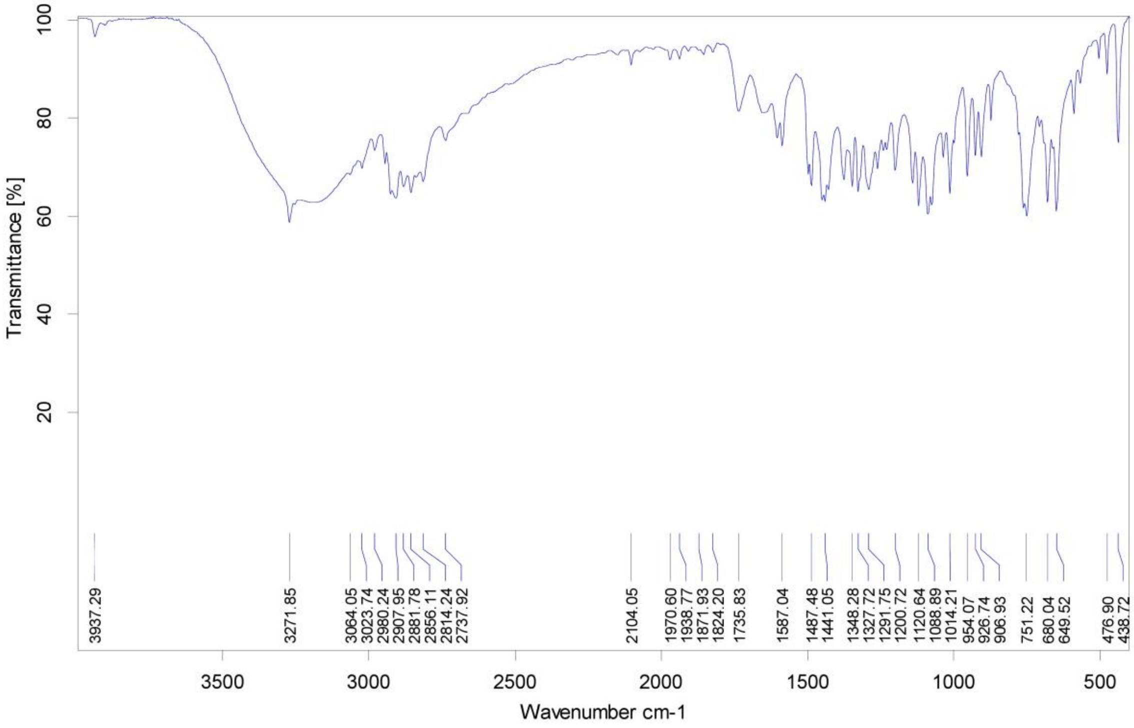

Figure S6 FT-IR spectrum of **5**

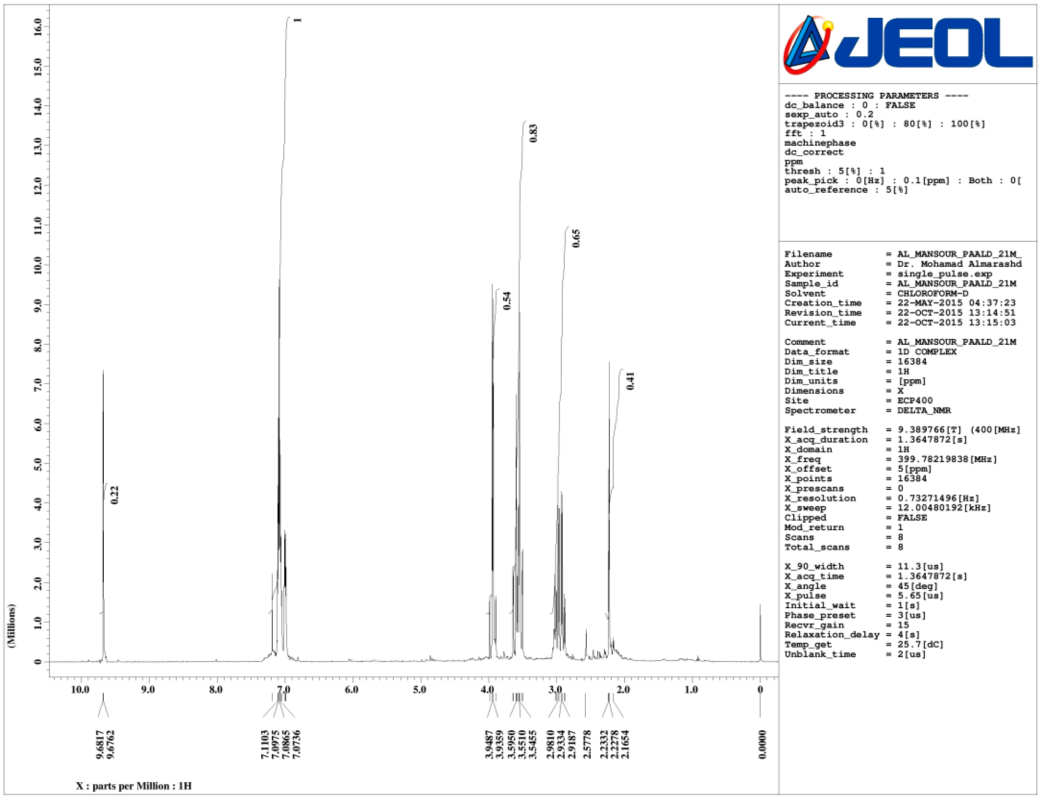

Figure S7 ^{1}H NMR spectrum of **6**

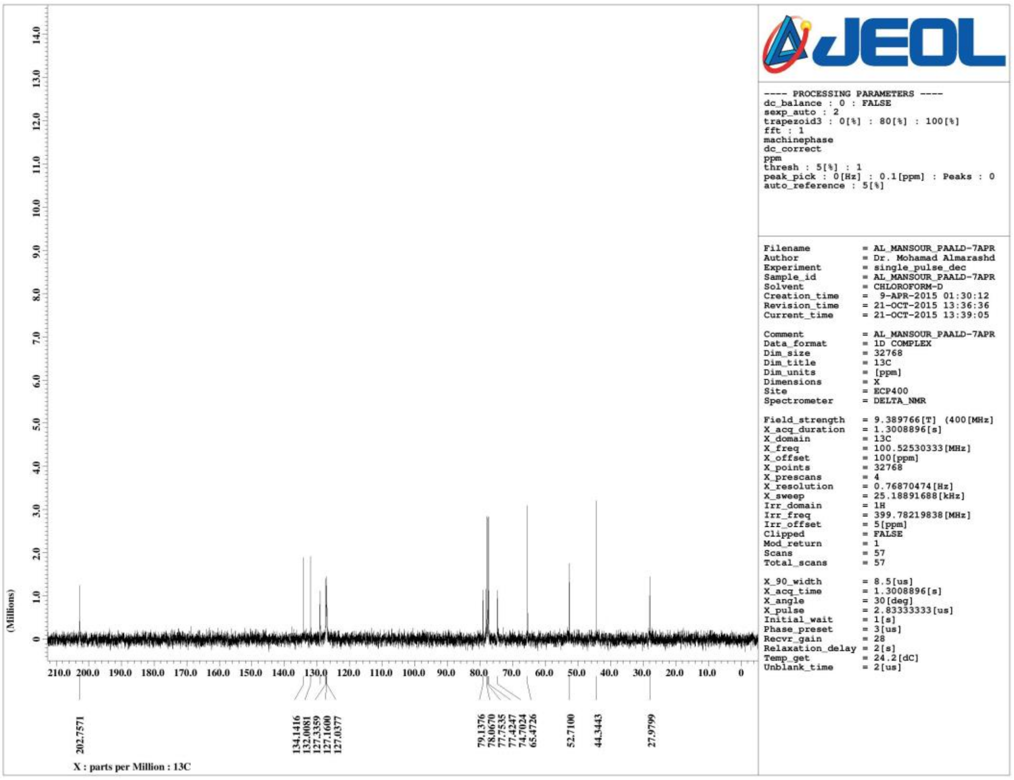

Figure S8 ^{13}C NMR spectrum of **6**

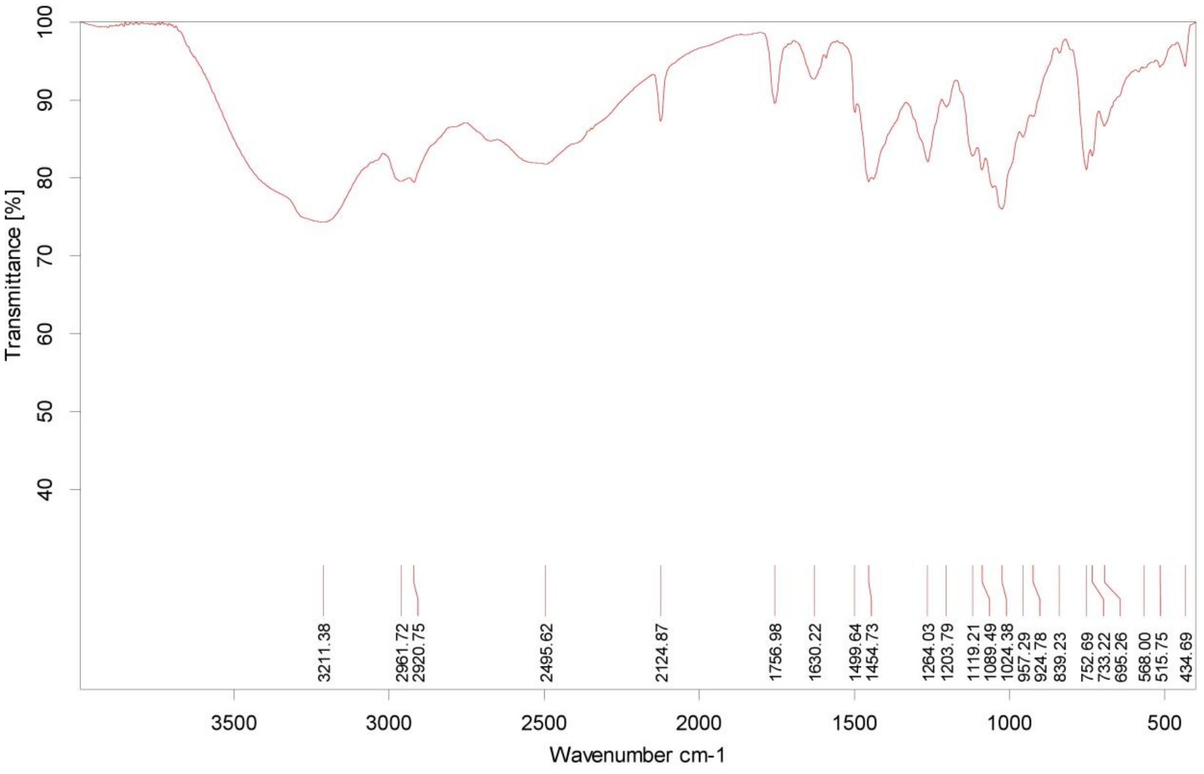

Figure S9 FT-IR spectrum of **6**

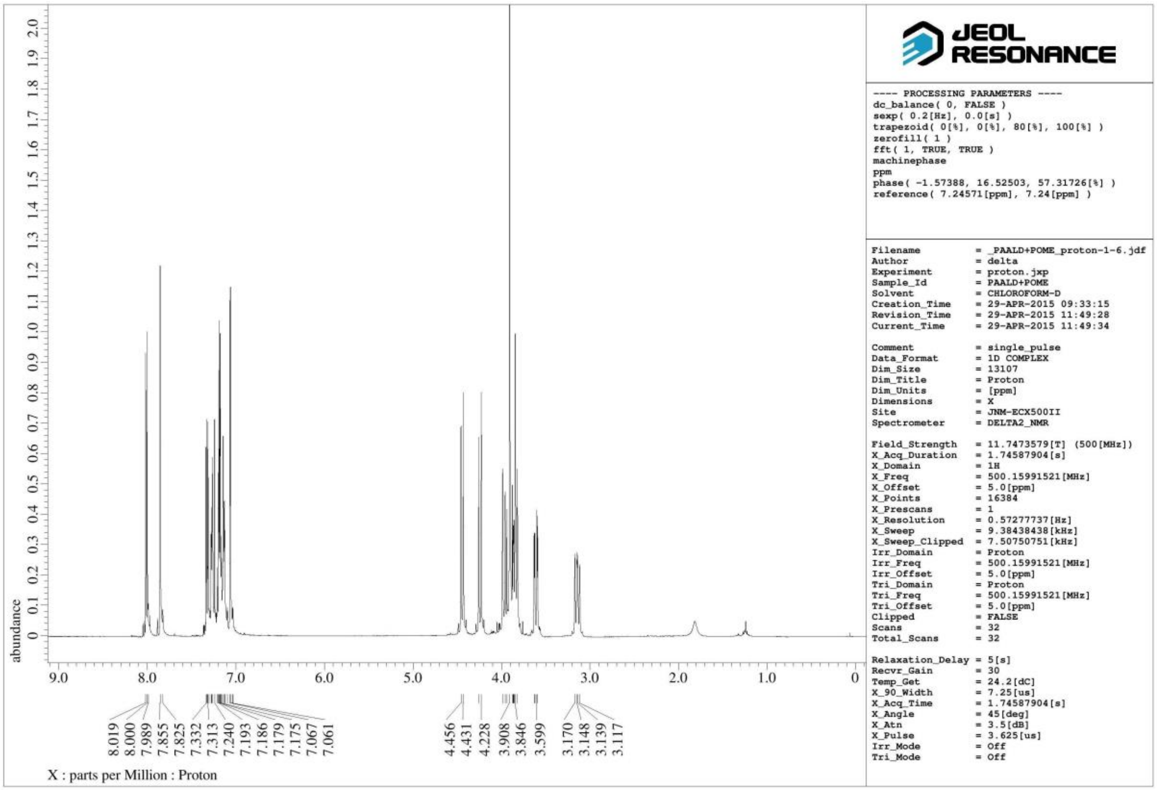

Figure S10 ^{1}H NMR spectrum of **8d**

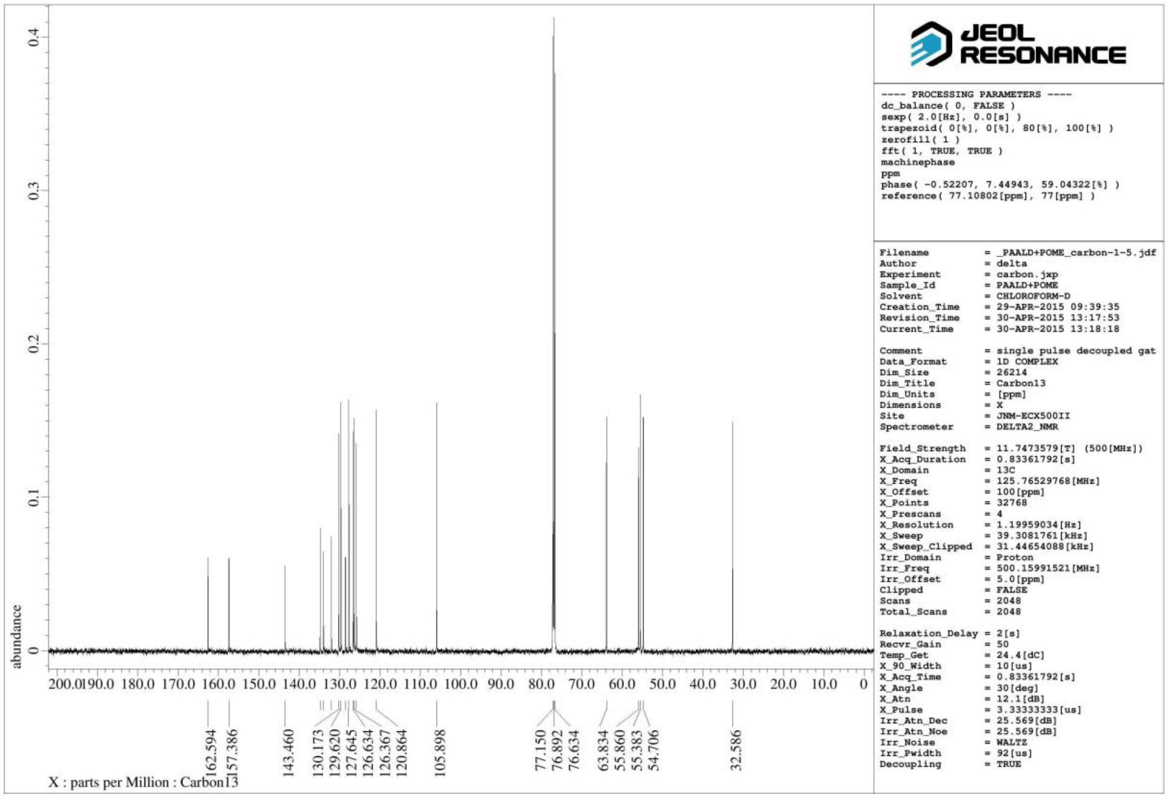

Figure S11 ^{13}C NMR spectrum of **8d**

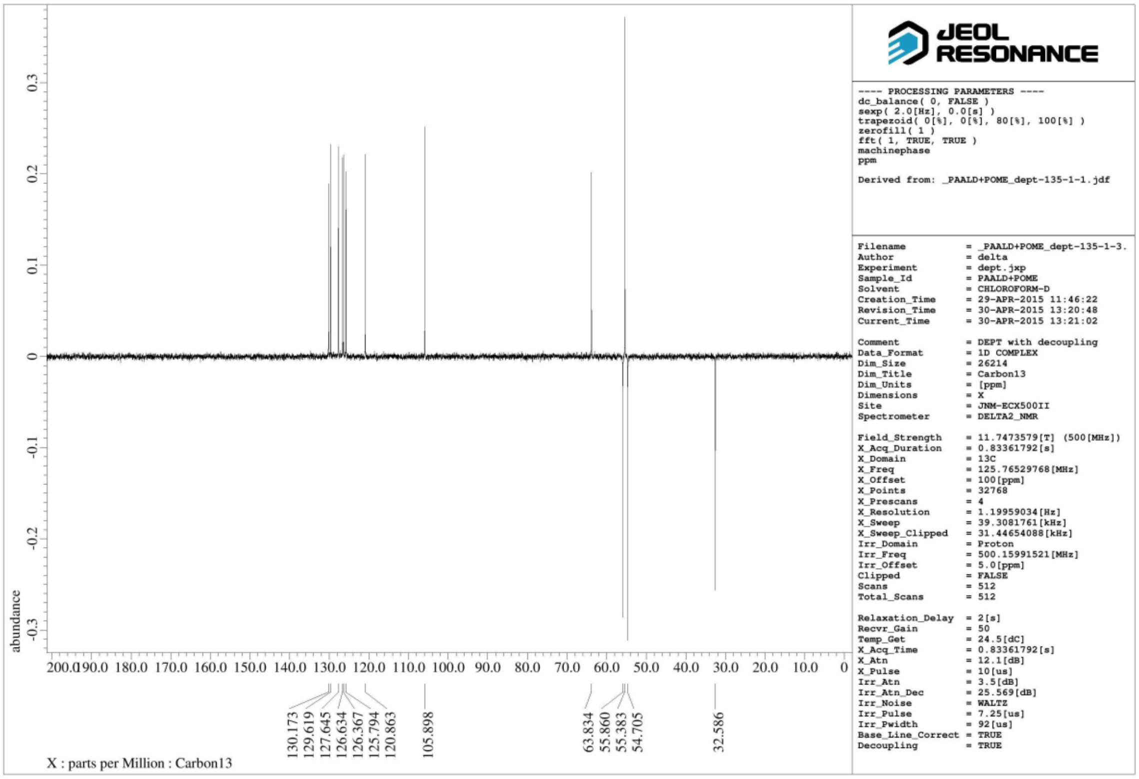

Figure S12 DEPT 135 NMR spectrum of **8d**

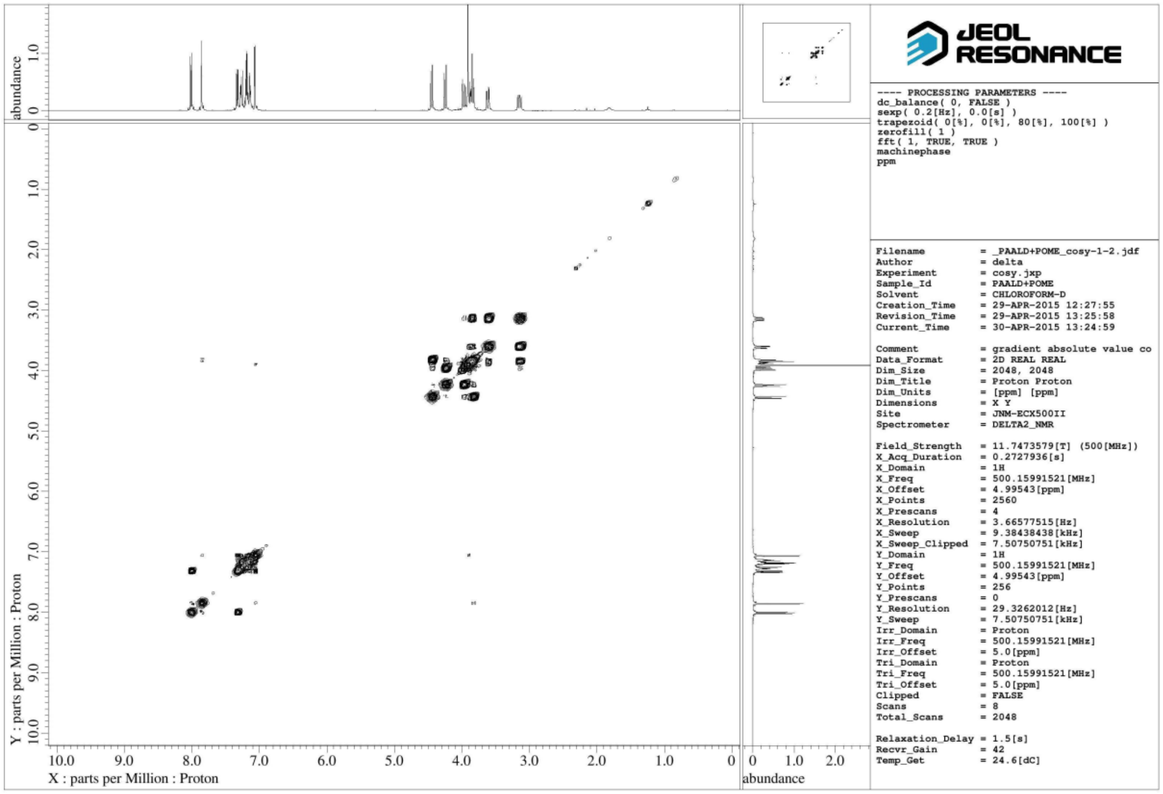

Figure S13 H-H COSY spectrum of **8d**

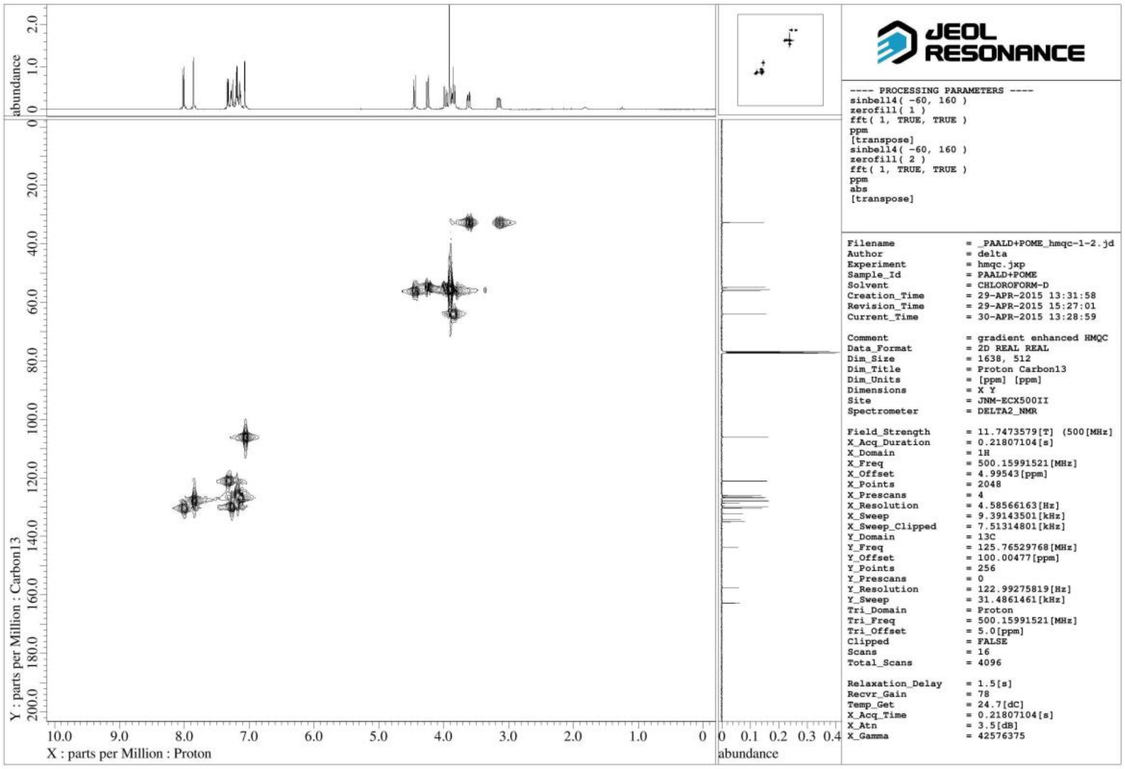

Figure S14 C-H COSY spectrum of **8d**

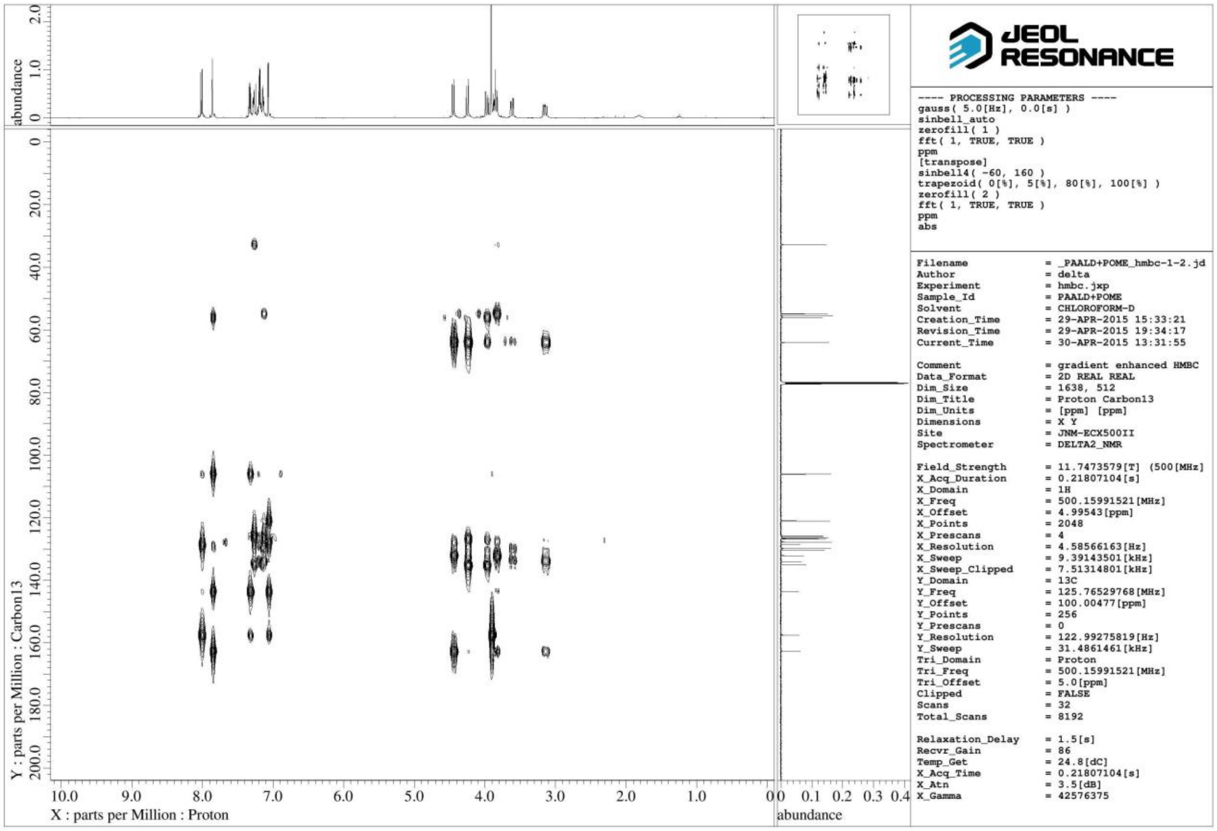

Figure S15 HMBC spectrum of **8d**

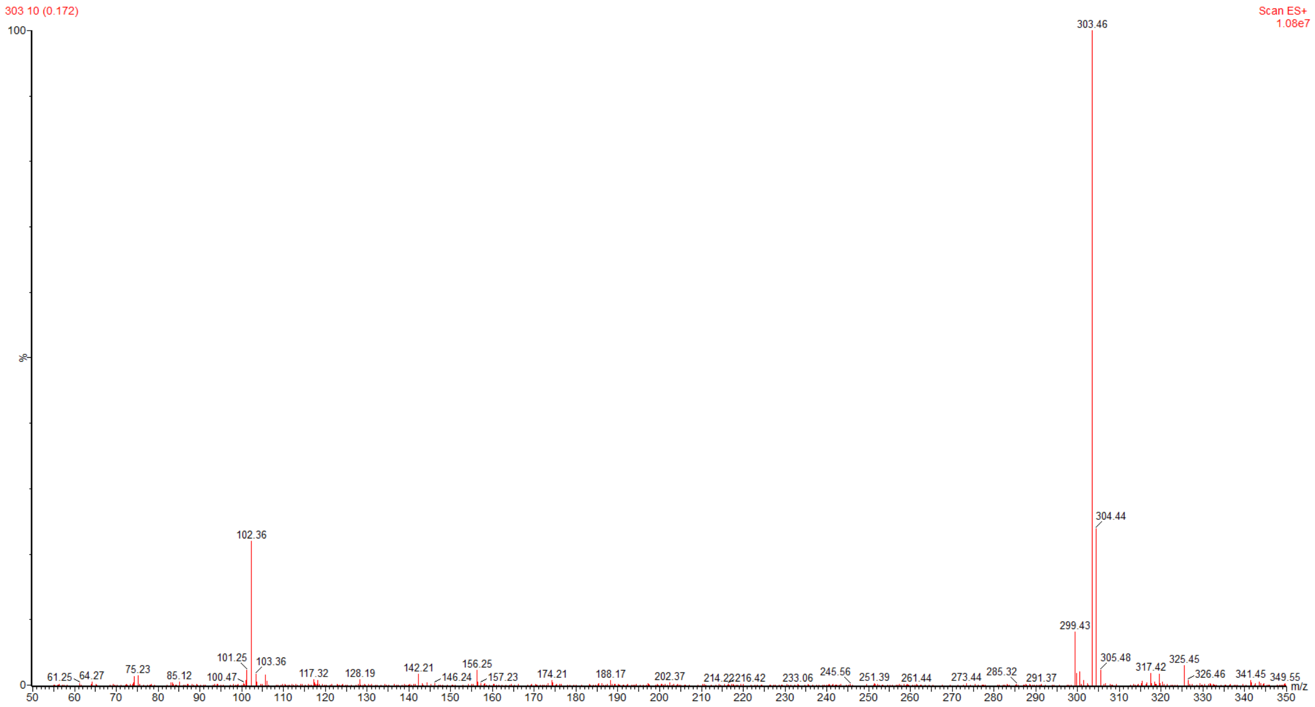

Figure S16 Mass spectrum of **8d**

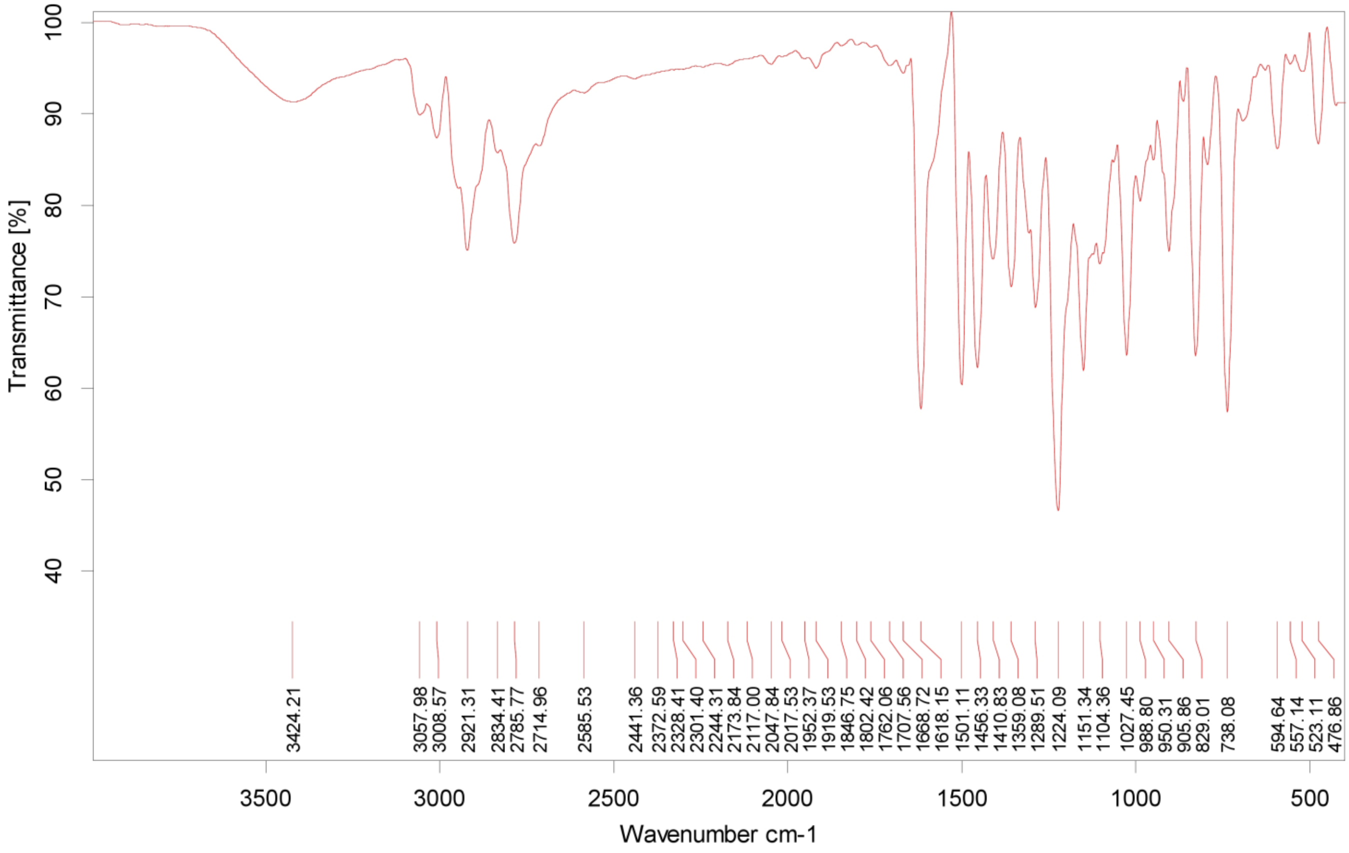

Figure S17 FT-IR spectrum of **8d**

Table S1 Experimental details

**Crystal data**	
Chemical formula	$C_{13}H_{15}NO$
Mr	201.26
Crystal system, space group	Orthorhombic, $P2_12_12_1$
Temperature (K)	150
a, *b*, *c* (Å)	6.5618 (3), 7.9332 (3), 21.0134 (9)
V (Å^3)	1093.88 (8)
Z	4
Radiation type	Mo *K*α
μ (mm^{-1})	0.08
Crystal size (mm)	0.67 × 0.42 × 0.21
**Data collection**	
Diffractometer	Bruker APEX-II D8 venture diffractometer
Absorption correction	Multi-scan SADABS Bruker 2014
Tmin, Tmax	0.94, 0.98
No. of measured, independent and observed [$I > 2\sigma(I)$] reflections	70825, 5294, 4966
R_{int}	0.031
**Refinement**	
$R[F^2 > 2\sigma(F^2)]$, $wR(F^2)$, S	0.033, 0.093, 1.04
No. of reflections	5294
No. of parameters	144
No. of restraints	0
H-atom treatment	H atoms treated by a mixture of independent and constrained refinement
$\Delta\rho_{max}$, $\Delta\rho_{min}$ (e Å^{-3})	0.30, −0.18

Table S2 Geometric parameters (Å, °)

O1—C10	1.4169 (12)	C6—C7	1.3977 (13)
O1—H1O1	0.84 (2)	C6—H6A	0.9300
N1—C11	1.4705 (13)	C7—C8	1.5022 (12)
N1—C1	1.4713 (11)	C8—C9	1.5223 (13)
N1—C9	1.4810 (12)	C8—H8A	0.9700
C1—C2	1.5026 (13)	C8—H8B	0.9700
C1—H1A	0.9700	C9—C10	1.5226 (12)
C1—H1B	0.9700	C9—H9A	0.9800
C2—C7	1.3948 (12)	C10—H10A	0.9700
C2—C3	1.3994 (12)	C10—H10B	0.9700
C3—C4	1.3891 (15)	C11—C12	1.4736 (15)
C3—H3A	0.9300	C11—H11A	0.9700
C4—C5	1.3912 (16)	C11—H11B	0.9700
C4—H4A	0.9300	C12—C13	1.1967 (16)
C5—C6	1.3891 (14)	C13—H13	0.99 (2)
C5—H5A	0.9300		
C10—O1—H1O1	107.8 (13)	C6—C7—C8	120.60 (8)
C11—N1—C1	109.22 (7)	C7—C8—C9	112.75 (7)
C11—N1—C9	113.40 (7)	C7—C8—H8A	109.0
C1—N1—C9	110.56 (7)	C9—C8—H8A	109.0
N1—C1—C2	113.40 (7)	C7—C8—H8B	109.0
N1—C1—H1A	108.9	C9—C8—H8B	109.0
C2—C1—H1A	108.9	H8A—C8—H8B	107.8
N1—C1—H1B	108.9	N1—C9—C10	112.53 (8)
C2—C1—H1B	108.9	N1—C9—C8	107.90 (7)
H1A—C1—H1B	107.7	C10—C9—C8	110.48 (7)
C7—C2—C3	119.33 (8)	N1—C9—H9A	108.6
C7—C2—C1	121.04 (7)	C10—C9—H9A	108.6
C3—C2—C1	119.59 (8)	C8—C9—H9A	108.6
C4—C3—C2	120.92 (9)	O1—C10—C9	108.85 (7)
C4—C3—H3A	119.5	O1—C10—H10A	109.9
C2—C3—H3A	119.5	C9—C10—H10A	109.9
C3—C4—C5	119.76 (9)	O1—C10—H10B	109.9
C3—C4—H4A	120.1	C9—C10—H10B	109.9
C5—C4—H4A	120.1	H10A—C10—H10B	108.3
C6—C5—C4	119.57 (9)	N1—C11—C12	114.27 (8)
C6—C5—H5A	120.2	N1—C11—H11A	108.7
C4—C5—H5A	120.2	C12—C11—H11A	108.7
C5—C6—C7	121.04 (9)	N1—C11—H11B	108.7
C5—C6—H6A	119.5	C12—C11—H11B	108.7
C7—C6—H6A	119.5	H11A—C11—H11B	107.6
C2—C7—C6	119.38 (8)	C13—C12—C11	177.86 (12)
C2—C7—C8	120.01 (8)	C12—C13—H13	175.1 (14)
